# A fungal endophyte of the medicinal plant, *Alisma orientale*, promotes plant growth and bioactive compound accumulation

**DOI:** 10.3389/fmicb.2026.1773907

**Published:** 2026-03-03

**Authors:** Xiaomei Xu, Wenjin Lin, Nemat O. Keyhani, Yamin Zhang, Junyong Han, Huiqing Que, Luxiao Wang, Yuhang Yao, Sen Liu, Xiaoyan Chen, Junzhi Qiu

**Affiliations:** 1 Fujian Key Laboratory of Medical Analysis, Fujian Academy of Medical Sciences, Fuzhou, China; 2 College of Biomedicine, Fujian Agriculture and Forestry University, Fuzhou, China; 3 Department of Biological Sciences, University of Illinois, Chicago, IL, United States; 4 State Key Laboratory of Agricultural and Forestry Biosecurity, College of Life Sciences, Fujian Agriculture and Forestry University, Fuzhou, China; 5 Key Laboratory of Traditional Chinese Medicine in Medical Institutions of Fujian Province, The Second Affiliated Hospital of Fujian University of Traditional Chinese Medicine, Fuzhou, China

**Keywords:** *Alisma orientale*, endophytic fungi, growth-promoting, triterpenoid, whole-genome sequencing

## Abstract

**Introduction:**

The Asian water plantain, *Alisma orientale* (Sam.) Juzep, is a flowering hydrophytic plant that grows in marshes. In traditional Chinese medicine, the rhizome of *A. orientale* is highly valued for its medicinal properties. Endophytic microbes modulate plant growth and the biosynthesis of secondary metabolites, however little is known concerning these effects in *A. orientale*.

**Methods:**

Here, high-throughput sequencing and culturing methods were utilized to investigate the endophytic fungal diversity in the rhizomes, flowers, roots and leaves of *A. orientale*. *In vitro* assays were employed to screen for strains exhibiting high growth-promoting abilities based on phosphate solubilization, siderophore production, oxidative stress resistance, and indole-3-acetic acid (IAA) production. Transcriptomics and whole genome sequencing were employed to investigate the underlying molecular mechanisms.

**Results:**

These data revealed that the Ascomycota and Basidiomycota were dominant phyla in all parts, with significant variation in fungal community composition observed at the genus level, as reflected in alpha and beta diversity indices. A total of 19 different endophytic fungal strains were isolated via culturing methods from the four different parts of *A. orientale*. *In vitro* assays resulted in the identification of four isolates subsequently used for co-culturing with sterile *A. orientale* to monitor plant-growth and terpenoid production. These latter results identified one promising strain, RT1, characterized as *Pseudothielavia terricola*. Isolate RT1 enhanced plant growth by 100–121% with respect to root length and plant height as compared to controls. After 21 days of treatment with strain RT1, the contents of the triterpenoids alisols B-23, C-23, and B were 4.5–5.5 times higher than those of the controls. Transcriptomics revealed enhanced expression of key enzymes involved in plant growth and bioactive compound accumulation, including 3-hydroxy-3-methylglutaryl-CoA reductase (HMGCR), mevalonate diphosphate decarboxylase (MVD), farnesyl diphosphate synthase (FPPS), 1-deoxy-*D*-xylulose-5-phosphate reductoisomerase (DXR), 1-deoxy-*D*-xylulose-5-phosphate synthase (DXS), farnesyl-diphosphate farnesyltransferase (FDFT1), and squalene monooxygenase (SQLE) during RT1 interaction. Whole genome sequencing of *P. terricola* revealed the presence of several gene clusters involved in tryptophan synthesis.

**Discussion:**

This study establishes endophytic fungal enhancement of *A. orientale* growth and bioactive compound accumulation, thereby increasing crop value and utility.

## 1 Introduction

*Alisma orientale* (Sam.) Juzep, known as “Zexie” in Chinese (common name: Asian water plantain), is a flowering hydrophytic plant that grows in marshes (Xu et al., 2024). It is highly valued in traditional Chinese medicine for its rhizomes, which are utilized in various compound formulations and Chinese patent medicines (Xu et al., 2020). Targeted research has revealed the beneficial effects of compounds derived from *A. orientale*, such as promoting diuresis, alleviating edema, reducing lipid levels, and displaying antiproliferative/anticancer properties. Hence, the plant is a valuable component of herbal remedies and a promising source for discovering bioactive compounds with diverse human health benefits/therapeutics (Feng et al., 2014; Zhang et al., 2017; Jang and Lee, 2021; Yan et al., 2022).

Triterpenoids are some of the most widely studied bioactive components and are responsible for many of the pharmacological effects of *A. orientale*. Triterpenoids encompass a large and diverse class of molecules typically composed of thirty carbon atoms and six isoprene units; these compounds are widely found in plants and marine organisms, and can contain tetracyclic and pentacyclic structures. In the 2020 version of the Chinese Pharmacopoeia, the protostane triterpenoids, alisol B-23 acetate and alisol C-23 acetate were designated as the quality control markers for *A. orientale,* reflecting the wide range of biotherapeutic activities attributed to these compounds (Gao et al., 2018). However, to date, nearly a hundred different triterpenoids have been isolated from *A. orientale* (Wang et al., 2020). Despite this rich diversity, their contents in harvested plants are relatively low, ranging from 0.004 to 0.114%, likely impacting the effectiveness of remedies. The triterpenoid content in *A. orientale* has been shown to vary significantly between different geographical locations, organs, and growth stages (Bailly, 2022; Tong et al., 2024). Thus, standardizing and/or promoting triterpenoid production and overall plant health holds significance.

Endophytic fungi colonize the tissues and organs of host plants asymptomatically during certain stages or throughout their life cycle, without causing any apparent symptoms of disease (White et al., 2019; Tshikhudo et al., 2023). Instead, these fungi establish mutualistic relationships with host plants, enabling nutrient exchange, which promotes plant health and resistance to abiotic and biotic stress. Many endophytic fungi hold crucial ecological roles by enhancing plant growth through activities such as nitrogen fixation, phosphate dissolution, siderophore production, and oxidative and other abiotic stress resistance, as well as protecting against plant pathogens (Liu et al., 2023; Qin et al., 2024). In addition, the interaction and exchange between these fungi and their plant hosts have been shown to promote the production of beneficial/protective secondary metabolites synthesized by either or both partners. These metabolites exert antimicrobial effects, and promote stress resistance, and plant/fungal growth; nonetheless, many biological functions remains obscure (Eshboev et al., 2023; Singh and Kumar, 2023; Waqar et al., 2024). Classic examples of fungal species from the *Mycena* genus colonizing orchids (*Dendrobium nobile* Lindl., *Gastrodia elata*) demonstrate that these fungi can regulate plant growth and development by secreting plant hormones, including indole acetic acid (IAA) (Zeng et al., 2018). In some instances, fungal pathogens have been reported to serve as beneficial endophytes. A strain of *Curvularia geniculate*, which is typically a plant pathogen, isolated from the roots of the Santa Maria feverfew, *Parthenium hysterophorus*, has been reported to promote plant growth by facilitating phosphorus solubilization and auxin (IAA) production (Priyadharsini and Muthukumar, 2017). Endophytes in tomatoes have also been shown to enhance plant growth and development by increasing the activity of antioxidant enzymes, such as catalase, superoxide dismutase, and peroxidase, and reducing cadmium accumulation (Gupta et al., 2023).

In addition to their growth-promoting properties, endophytic fungi are recognized for their significant role in promoting and contributing to the synthesis and accumulation of secondary metabolites within host plants. Some endophytic fungi produce metabolites similar to or even identical to those produced by their host plants. Fungal endophytes can also positively affect the accumulation of specific active compounds in many medicinal plants, the most common of which are terpenoids, alkaloids and polyphenols, which represent the largest classes of such molecules (Prabha et al., 2018; Gao et al., 2025). Endophytic fungi influence the biosynthesis of terpenoid indole alkaloids (TIAs), which are compounds with notable therapeutic properties, including antimicrobial, anti-inflammatory, and/or antiproliferative (anticancer) activities (Khalkho et al., 2024). Notably, colonization by the endophytic fungus *Pseudodidymocyrtis lobariellae* KL27 has been shown to increase taxol accumulation by 3.2 times in the host plant *Taxus chinensis* compared to non-colonized plants. Taxol is an extensively studied compound with anticancer properties (Cao et al., 2022). Furthermore, fungi can serve as an important contributor to compound diversity and discovery. In some, instances, the fungus can be an important contributor to compound diversity and discovery. For example, an endophytic fungus isolated from the Caspian sage (*Salvia abrotanoides*) has been shown to produce cryptotanshinone, a bioactive compound that inhibits STAT3 signaling. Cryptotanshinone exhibits anti-proliferative, anti-inflammatory, and cardiovascular protective activities. These findings highlight the potential of endophytic fungi as a source of novel bioactive compounds with applications in pharmaceutical and agricultural development (Teimoori-Boghsani et al., 2019). Despite the well-documented benefits of endophytes in promoting plant growth, research on isolating endophytic fungi from *A. orientale* with similar growth-promoting properties is limited.

The primary objectives of this study were to investigate the endophytic fungal diversity in *A. orientale* and to screen the plant growth-promoting properties of these endophytes, as well as their impact on the accumulation of beneficial triterpenoids. High-throughput sequencing and traditional culture methods were utilized to investigate the endophytic fungal diversity in different plant parts of *A. orientale*. A set of endophytes with growth-promoting potential was identified via assays for assessing phosphate solubilization, siderophore production, oxidative stress resistance, and IAA production. The identified growth-promoting endophytic fungi were further co-cultivated with sterile seedlings of *A. orientale* and transcriptomic and UPLC-MS analyses were conducted to explore plant responses and the underlying molecular mechanisms for growth promotion and the accumulation of triterpenoids. In addition, whole-genome sequencing was used to provide insights into the genetics affecting the production of triterpenoid compounds by the endophytic fungus.

## 2 Materials and methods

### 2.1 Plant sampling

**Figure 1:**
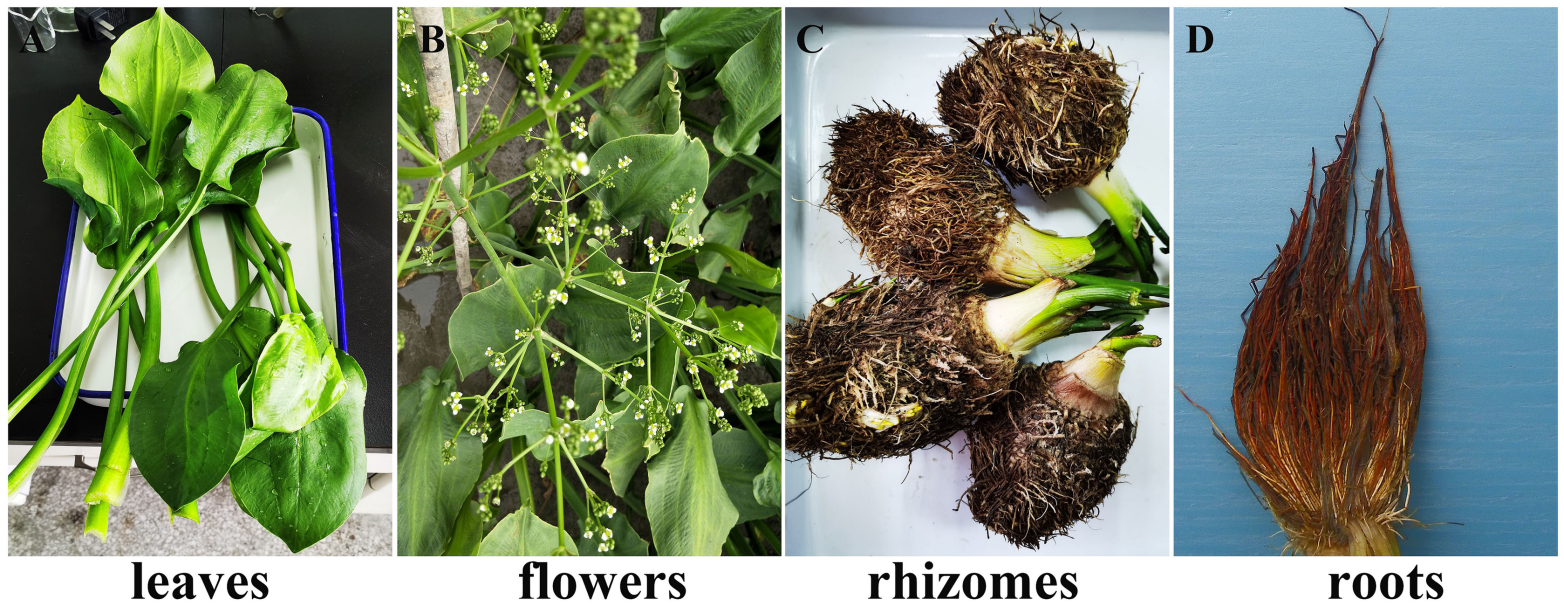
Morphology of *A. orientale* leaves **(A)**, flowers **(B)**, rhizomes **(C)** and roots **(D)** of *A. orientale*.

Fresh samples of *A. orientale* were collected from Jian′ou County, Fujian Province, China (27°18′18′′ N, 118°8′21′′ E, in 2022) and separated into four different parts: leaf, flower, rhizome and root (Figure 1). All collected samples were kept on ice and transported to the laboratory for further processing.

### 2.2 Isolation and identification of fungal endophytes from *A. orientale*

Fungal endophytes were isolated from the different plant parts within 24 h of harvest. Surface sterilization of the plant samples was performed according to previously described methods (Rehman et al., 2022; Shen et al., 2023). Briefly, separated plant parts (rhizomes, flowers, roots and leaves) were rinsed repeatedly with running water to remove surface soil particles and adhered debris. Sections were then sliced into smaller sections and moved to a clean bench for surface sterilization as follows: samples were rinsed in sterile water for 30 s, then soaked in 75% ethanol for 2 min, followed by a 5 min exposure to 3% NaClO, and 3X rinses with sterile water and then drained on sterile filter paper. Samples were then cut into 2 mm^3^ sections using a sterile knife. A portion of the disinfected samples were immersed in liquid nitrogen for 20 min and then stored at −80 °C for future use (high-throughput sequencing). The remaining portions were used for isolation of endophytic fungi as follows: sections of cut tissue pieces were placed on (i) potato dextrose agar (PDA) media, (ii) Rose Bengal agar (RBA), and (iii) corn meal malt extract agar (CMM) supplemented with 50 mg/L chloramphenicol to inhibit the growth of bacteria. The plates were cultured at 28 °C for 7–10 days. Any fungal colonies apparent were then subculture by transferring the hyphal tips to fresh culture dishes until colonies appeared pure. The pure isolated strains were stored in 15% glycerol (v/v) at −20 °C (Chowdhary and Sharma, 2020).

A total of 19 strains were purified from four different parts of *A. orientale*, comprising 6 strains from rhizomes, 5 from roots, 5 from leaves, and 3 from flowers, designated as RH1-6, RT1-5, LF1-5, and FL1-3, respectively. Fungal isolates were subsequently identified by morphology and DNA loci sequencing. Fungal genomic DNA isolation was purified using Fungal DNA Kit (Omega Bio-tek, Inc.) according to the manufacturer’s instructions. Primers pair used for amplification of target genes were listed in Supplementary Table S1 and included ITS, LSU, IGS, TEF, CAM, RPB1, RPB2, TUB2, ACT, CMD, GAPDH, HIS, TEF1-*α*, and BenA loci. PCR products from genomic loci amplification were sent to Sangon Biotech (Shanghai) Co., Ltd. for sequencing. Sequences were aligned with the NCBI GenBank database using BLAST to identify those with high similarity. Clustal X 1.83 software was used to carry out multiple sequence alignments. Phylogenetic analysis was conducted using MEGA X via the Neighbor-Joining (NJ) method with 1,000 bootstraps.

### 2.3 High-throughput sequencing of endophytic fungi

After surface disinfection, plant section samples frozen in liquid nitrogen and stored at -80 °C were sent to Gene *De novo* Biotechnology Co., Ltd. (Guangzhou, China) for ITS amplicon high-throughput sequencing analysis using the primers ITS1_F_KYO2 (TAGAGGAAGTAAAAGTCGTAA) and ITS86R (TTCAAAGATTCGATGATTCAC). Three biological replicates were included for each of the experimental groups.

### 2.4 Qualitative and quantitative analysis of growth-promoting characteristics

#### 2.4.1 IAA production

Indole-3-acetic acid (IAA) production was measured according to Gordon and Weber (1951). Briefly, fungal isolates were inoculated in King’s media (50 mL) with or without L-tryptophan at 28 °C for 10 days. After incubation, mycelia were removed by centrifugation at 10,000 rpm/min for 10 min. An aliquot of the cell-free supernatant (0.5 mL) was mixed with an equal volume of Salkowski reagent. For qualitative estimation, a pink color change signified a positive result, with no color change, negative. For quantitative estimation, after mixing the Salkowski reagent with the supernatant in equal volume, the mixture was incubated in the dark for 30 min, and the OD_530_ value was measured. The concentration of IAA produced by the fungal strains was estimated using a standard curve.

#### 2.4.2 Phosphate solubilization

Phosphate solubilization was done on National Botanical Research Institute Phosphate (NBRIP) solid medium supplemented with Ca_3_PO_4_. After incubation at 28 °C for 5–7 days, the presence of transparent circles around growing colonies was to determine phosphorus solubilization activity. Endophytic fungal isolates identified in the qualitative estimation were subsequently individually cultured in Pikovskaya (PVK) media. Following 10 days of incubation at 28 °C with constant shaking of 120 rpm/min in darkness, the filtrate was collected after centrifugation at 10,000 rpm/min for 10 min. The cell-free supernatant was subjected to quantitative phosphorus analysis using the Phosphorus (Pi) Colorimetric Assay Kit (Elabscience Biotechnology Inc.) (Liu et al., 2020). Eight concentrations of phosphorus standard solutions (0, 0.1, 0.2, 0.5, 0.8, 1.0, 1.5 and 2.0 mmol/L) were used to develop a standard curve. Medium without fungal inoculation served as the control. After preparing the supernatant of the samples and standard solutions according to the instructions, incubated at 37 °C for 30 min and the OD_660_ value was measured for each well. Performed triplicates for each sample. The phosphate concentration in the samples was calculated based on the standard curve.

#### 2.4.3 Siderophore production

For siderophore production, fungal isolates were cultured on CAS blue agar medium for 3–5 d. Formation of an orange halo around colonies was used to qualitatively measure siderophore production (Deng et al., 2022). For quantitative estimation, the CAS detection solution was mixed with an equal volume of the cell-free supernatant from cultures as above in equal volume, the mixture was incubated in the dark for 30 min, and the OD_630_ value (As) was measured. The absorbance of uninoculated culture medium mixed with CAS detection solution was used as the reference value (Ar). The siderophore production index was calculated as SU = [(Ar−As)/Ar] × 100%, where SU represents the units of iron carrier activity.

#### 2.4.4 Catalase (CAT) activity

Fungal isolates were inoculated in PDB medium and grown at 28 °C with aeration (120 rpm/min) in darkness for 10 days. The cell-free culture supernatant was collected after centrifugation at 10,000 rpm/min for 10 min and assayed for catalase (CAT) activity using the Catalase (CAT) Activity Assay Kit as described (Elabscience Biotechnology Inc.) (Liu et al., 2021). Eight concentrations of H_2_O_2_ standard solutions (0, 10, 20, 30, 40, 50, 60 and 100 μmol/mL) were used to develop a standard curve. Medium without fungal inoculation served as the control. Measurement of the yellowish complex generated by the interaction of ammonium molybdate with peroxide was conducted at 405 nm. The catalase activity of the samples was calculated based on the standard curve. Three replicates should be performed for each sample.

### 2.5 Preparation of *A. orientale* sterile seedlings and co-culture experiments

*A. orientale* seeds were rinsed thoroughly with tap water to remove surface soil particles and soaked in water for 3 h before undergoing surface sterilization as follows: seeds were rinsed in sterile water for 30 s, then soaked in 75% ethanol for 2 min, followed by a 15 min exposure to 3% NaClO. Seeds were then rinsed three times with sterile water and then dried on sterile filter paper. The sterilized seeds were placed in tissue culture bottles containing Murashige and Skoog (MS) media for germination, and incubated in an illuminating incubator protected from light at 28 °C for 7 d. After germination, the seedlings were subjected to a 14 h light/10 h dark photoperiod at 28 °C and an illumination intensity of 6,000 Lux. After one week of light exposure, seedlings were transferred for continued growth, with three seedlings/bottle under the same lighting conditions. Cultivation was continued for an additional three weeks. Co-culture of plant with fungal inocula was performed by placing a 5 mm diameter fungal agar plug from a growing plate 3 cm from the root tip of the seedling. Each seedling was inoculated with one fungal agar block. Control (uninoculated) PDA plugs were used as negative controls treatments. After three weeks of co-culture, plant root length, height, lateral root number, fresh weight, and number of leaves were recorded immediately after harvest, whereas total dry weight was measured from samples after 8 h oven drying at 50 °C to constant weight.

The main roots of *A. orientale* in different groups were collected after three weeks of co-culture. The roots intended for scanning electron microscopy (SEM) were fixed in 2% glutaraldehyde at 4 °C for 24 h, followed by three washes with 0.1 M phosphate buffer. Subsequently, the samples were fixed in 1% osmium tetroxide in 0.1 M phosphate buffer at room temperature in the dark for 2 h. Post-fixation, the root tissues were dehydrated in a series of ethanol gradients (30, 50, 70, 80, 90, 95, and 100%) for 15 min each. Following dehydration, the roots were dissected with a sterile surgical blade, dried overnight in a desiccator, coated with a gold–palladium sputter, and then observed and imaged using SEM with the internal structure facing upwards.

### 2.6 Transcriptome sequencing and RT-qPCR verification analysis

*A. orientale* plants were grown with or without endophytic fungal strain RT1 (identified as *Pseudothielavia terricola*) for 21 days as described above. Total RNA was extracted from the whole plant (~ 0.5 g) using the Omega Plant RNA Kit (Omega Bio-tek, Inc.) according to the manufacturer’s instructions. Samples were analyzed by 1% agarose gel electrophoresis to evaluate RNA integrity, and RNA purity and concentration were measured using a NanoDrop Spectrophotometer. Library construction and transcriptome sequencing were completed by Gene *Denovo* Biotechnology Co., Ltd. (Guangzhou, China). Differentially expressed genes (DEGs) were analyzed using the DESeq2 software with the screening criteria set as |log2foldchange| ≥ 1 and *p* < 0.05. GO (Gene Ontology; http://geneontology.org) and KEGG (Kyoto Encyclopedia of Genes and Genome; http://www.genome.jp/kegg) enrichment analysis were used for further analysis of the DEGs. The Blast2GO (version 2.5.0) was used to obtain the GO annotations of unigenes. Sequences in KEGG databases were searched using Blastx.

Real-time quantitative PCR (RT-qPCR) was used to evaluate the expression levels of five DEGs involved in plant growth promotion and triterpenoid accumulation. Expression of the *UBC9* gene was employed as the reference. The primer pairs used for PCR amplification of target genes are listed in Supplementary Table S2. The relative gene expressions were calculated using the 2^-ΔΔCt^ method. RT-qPCR analyses were performed using three biological replicates with technical duplicates.

### 2.7 UPLC-MS measurement of triterpenoids content

The contents of triterpenoids, including alisol B, alisol B-23 acetate, and alisol C-23 acetate, were quantified via UPLC-MS. To determine triterpenoid content, 0.5 g of plant dried powder from the treatment and control groups was dissolved in 25 mL of acetonitrile and subjected to ultrasonic extraction for 30 min. Prior to UPLC-MS analysis, the solution was filtered using a 0.22 μm microporous membrane filter. A calibration curve was established using standards purchased from Chengdu Must Biotechnology Co., Ltd., which were processed under identical conditions to the samples. Three replicates were prepared for each group.

### 2.8 Whole genome sequencing analysis

Total genomic DNA of the fungus strain RT1 (*Pseudothielavia terricola*) was extracted using QIAGEN Genomic-tip 20/G kit and analyzed by 0.5% agarose gel electrophoresis to evaluate the integrity of DNA. The purity and concentration of DNA were measured with the NanoDrop 2,100. Whole genome sequencing was carried out by Biomarker technologies Co., Ltd. (Beijing, China) on a PacBio Sequel II platform with one SMART cell, which generated long-read data.

### 2.9 Statistical analysis

Results were shown as mean ± SD based on three independent tests. ANOVA (analysis of variance) was used to compare differences between experimental groups, followed by the Student Newman–Keuls test (SNK). Statistical significance was determined by *p* < 0.05.

## 3 Results

### 3.1 Endophytic fungal diversity in *A. orientale*

**Figure 2:**
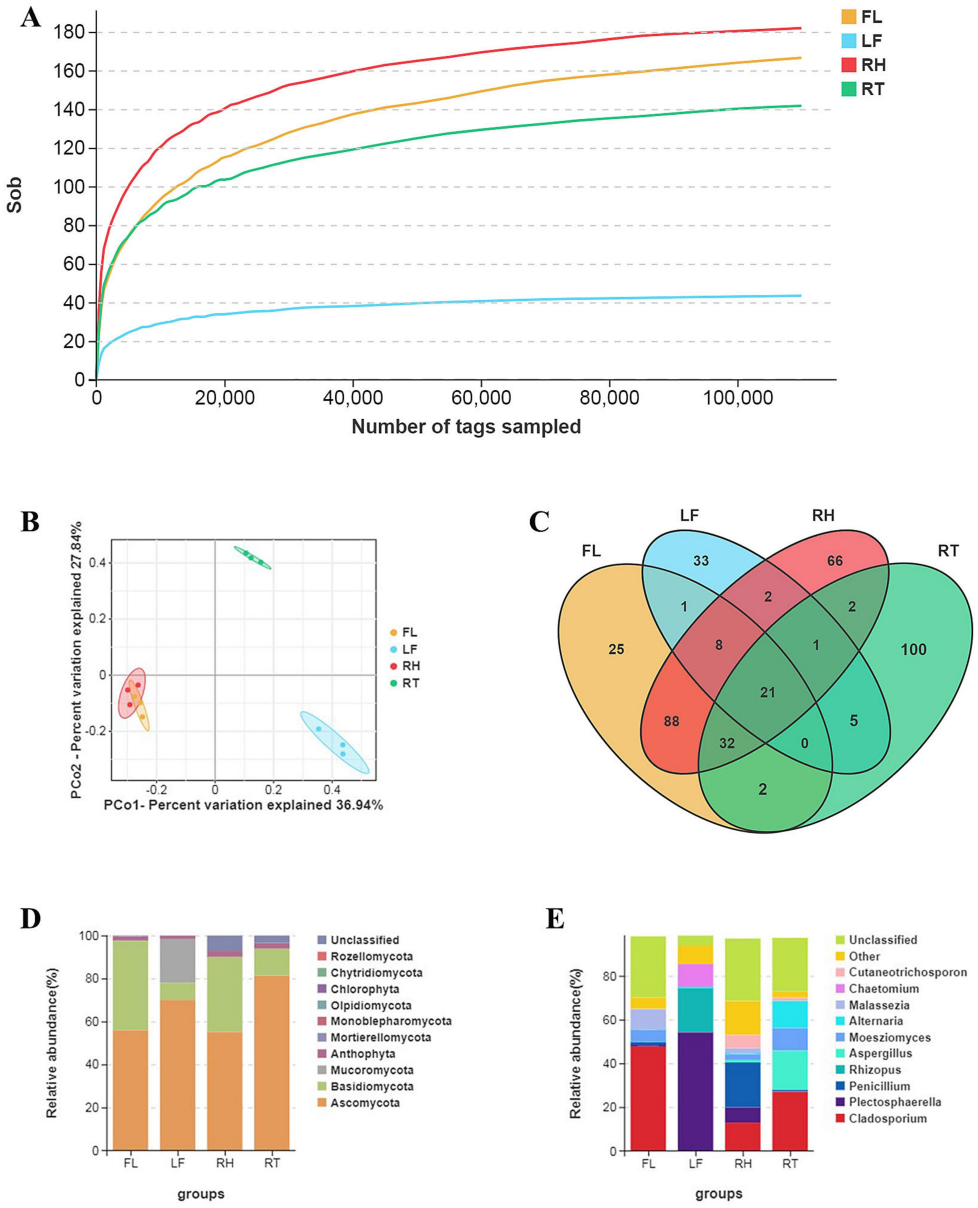
Endophytic fungal diversity in *A. orientale*. **(A)** ASV rarefaction curves for different parts of *A. orientale*. **(B)** Venn diagram showing the number of ASVs shared and specific for different parts of *A. orientale*. **(C)** PCoA analysis of endophytic fungi in *A. orientale* based on beta diversity index. **(D)** Relative abundance of phyla in each group (top 10). **(E)** Relative abundance of genus in each group (top 10).

The endophytic fungal diversity of four different parts of *A. orientale*, including rhizomes (RH), flowers (FL), roots (RT), and leaves (LF), was determined by high-throughput sequencing. A total of 489,043 raw reads were obtained. Following assembly and chimeric filtering, 484,139 effective tags were retained, resulting in a high data validity rate of 94% (Table 1). The average sequence length was 333 bp. The ASV rarefaction curves are shown in Figure 2A. The saturation observed in the four sample groups confirmed that the sequencing data depth could fully reflect the richness and diversity present in the current samples.

**Table 1:** The data of high-throughput sequencing analysis for different parts of *A. orientale.*

Sample name	Raw reads	Clean reads	Raw tags	Clean tags	Chimera	Effective tags	Effective ratio (%)
FL	131,066	130,839	127,691	126,786	327	126,460	96.49
LF	129,423	129,356	119,243	118,167	37	118,129	91.39
RH	127,373	127,172	119,773	118,981	756	118,225	92.84
RT	129,538	129,045	122,336	121,780	455	121,325	93.64

A total of 629 ASVs were identified across four parts of the *A. orientale*, with 177, 71, 218, and 163 ASVs found in the FL, LF, RH and RT, respectively (Figure 2B). Among these, 21 ASVs were shared among all four groups, while the FL, LF, RH and RT groups had 25, 33, 66, and 100 unique ASVs, respectively. Alpha-diversity analysis of different parts of *A. orientale* indicated that fungal communities richness and diversity was highest in *A. orientale* rhizome samples, followed by flowers and roots, with the lowest diversity found in the leaves (Table 2). However, beta-diversity analysis showed that groups RH and FL were separate from the RT and LF samples, indicating a high similarity of the endophytic fungal communities between the groups of RH and FL. The endophytic fungal communities in the roots and leaves exhibit significant differences from the former two groups (Figure 2C).

**Table 2:** Statistical analysis of alpha diversity indices of endophytic fungi in *A. orientale.*

Groups	ACE	Chao1	Shannon	Simpson
FL	183.70 ± 10.58^a^	182.64 ± 5.88^a^	2.53 ± 0.33^a^	0.68 ± 0.09^a^
LF	46.05 ± 12.96^b^	45.32 ± 11.56^b^	1.40 ± 0.17^b^	0.43 ± 0.09^b^
RH	192.70 ± 11.19^a^	191.03 ± 9.54^a^	4.05 ± 0.53^c^	0.88 ± 0.05^c^
RT	155.97 ± 11.42^c^	152.51 ± 9.45^c^	2.95 ± 0.87^ac^	0.73 ± 0.12^ac^

Data were presented as the means ± SD. Different letters in the same column indicated statistical significance (*p* < 0.05).

Based on the annotation of ASVs and the analysis of relative abundance, a total of 8 phyla, 24 classes, 48 orders, 86 families, and 111 genera of endophytic fungi were identified in the four different parts of *A. orientale*. As expected, Ascomycota and Basidiomycota were the dominant phyla in all four groups, with relative abundances ranging from 55.18 to 81.26% and from 7.97 to 41.53%, respectively (Figure 2D). For screening key differential genera, the top 10 genera in terms of abundance in the four groups were selected (Figure 2E). The dominant genera in the FL group were *Cladosporium* (47.67%), *Malassezia* (9.16%) and *Moesziomyces* (5.38%), with *Cladosporium* being the most abundant genus. *Plectosphaerella* (54.12%), *Rhizopus* (20.27%) and *Chaetomium* (10.27%) were the dominant genera in leaves. The dominant genera in the RH group were *Penicillium* (20.58%), *Cladosporium* (12.93%) and *Plectosphaerella* (6.91%). The dominant genera in the RT group were *Cladosporium* (26.99%), *Aspergillus* (17.97%), *Alternaria* (12.44%) and *Moesziomyces* (10.20%). These results indicated that the fungal community composition in different parts of the *A. orientale* varied greatly at the genus level.

### 3.2 Culturing and identification of endophytic fungi from *A. orientale*

**Figure 3:**
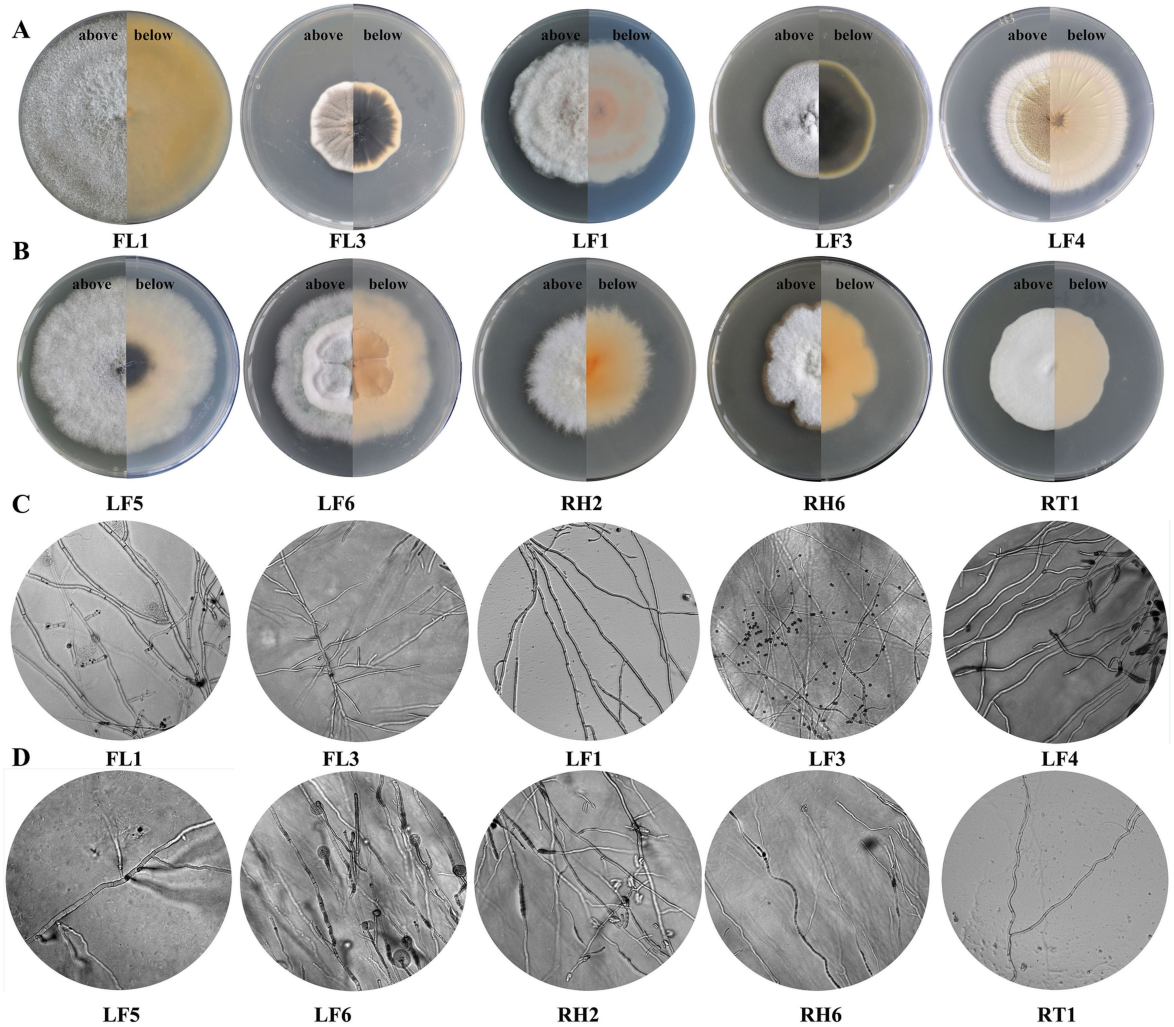
Morphology of specific endophytic fungi. **(A)** and **(B)** colony morphology as seen from above and below, respectively; **(C)** and **(D)** Morphological characteristics of the hyphae of specific endophytic fungi on PDA (20x).

A total of 19 endophytic fungi were isolated from the four different parts of *A. orientale* by culturing sterile sections on PDA as detailed in the Methods section. These included 6, 5, 5 and 3 strains from the rhizomes (RH1-6), roots (RT1-5), leaves (LF1-5), and flowers (FL1-3), respectively. All strains were initially identified according to their morphological characterizations (Figure 3) combined with molecular sequence analyses of ITS/LSU genes. Moreover, in conjunction with the relevant fungal genera, the genes were further analyzed for species classification (Supplementary Table S3). The closest identified matches are listed in Table 3. The nucleotide sequences of 19 endophytic fungi exhibited a similarity of over 98% to the most closely related sequences in the nucleotide database. The phylogenetic tree of endophytic fungi can be found in Supplementary Figure S1. All 19 endophytic fungi isolates belonged to the phylum Ascomycota and were distributed across 3 classes, 5 orders, 8 families, and 9 genera. The most common isolated fungi were *Penicillium* sp. (6 isolates, all corresponding to *P. oxalicum*), followed by *Nigrospora* sp. (3 isolates, two corresponding to *N. sphaerica,* and one *N. oryzae*), and *Fusarium* sp. (3 isolates, all *F. proliferatum*).

**Table 3:** Identification of fungal isolates via ITS/LSU sequence comparison.

ID	Genus	Accession number	Identity (%)
FL1	*Nigrospora sphaerica*	MW081353	99.82
FL2	*Nigrospora sphaerica*	KU204768	99.82
FL3	*Cladosporium* sp.	MF473304	99.82
LF1	*Pestalotiopsis microspora*	MK432981	100.00
LF2	*Penicillium oxalicum*	KT355727	99.66
LF3	*Cercospora* sp.	MK752900	99.81
LF4	*Aspergillus tubingensis*	KY552633	99.66
LF5	*Nigrospora oryzae*	KX219600	99.82
RH1	*Fusarium proliferatum*	MF471668	100.00
RH2	*Fusarium proliferatum*	JQ690083	100.00
RH3	*Fusarium proliferatum*	OQ555139	100.00
RH4	*Penicillium oxalicum*	OQ629128	99.50
RH5	*Cladosporium* sp.	KX078479	99.82
RH6	*Talaromyces trachyspermus*	MT529356	100.00
RT1	*Pseudothielavia terricola*	KX431226	99.82
RT2	*Penicillium oxalicum*	KX067861	100.00
RT3	*Penicillium oxalicum*	MN856284	99.66
RT4	*Penicillium oxalicum*	MN856268	100.00
RT5	*Penicillium oxalicum*	MN856284	99.66

### 3.3 Growth-promoting characteristics of endophytic fungi

**Figure 4:**
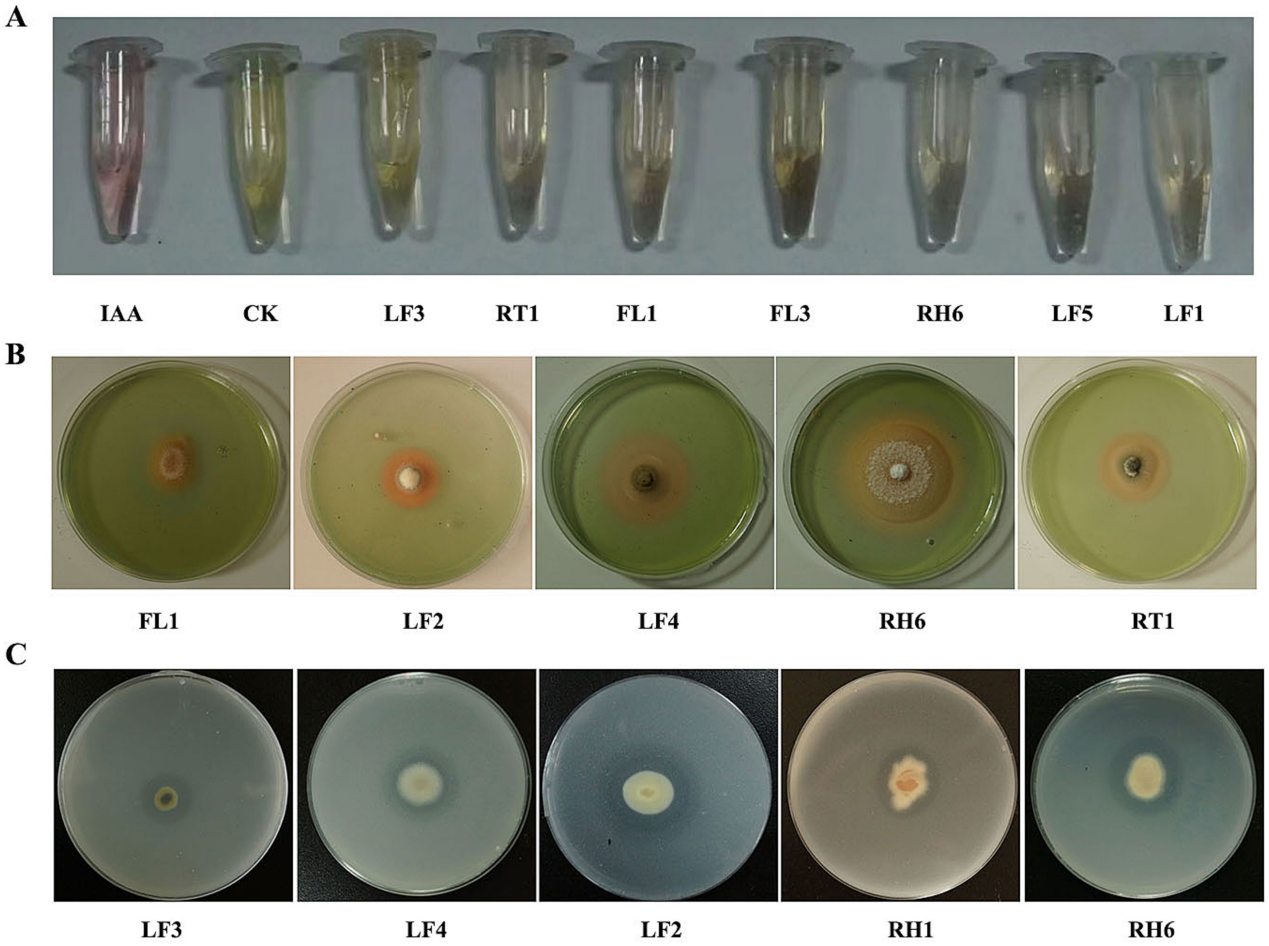
Qualitative detection of growth promoting abilities of endophytic fungi. **(A)** IAA production; **(B)** siderophore production; **(C)** phosphate solubilization.

Fungal isolates were initially screened qualitatively, and then quantitatively for traits associated with plant growth promotion and/or enhancement of secondary metabolite production, as detailed in the Methods section. The results revealed seven IAA-producing strains, five strains capable of promoting phosphate solubilization, five strains positive for siderophore production, and six strains exhibiting antioxidant (catalase) activity (Figure 4; Table 4). Fungal isolates FL1 (*Nigrospora sphaerica*) and RT1 (*Psedothielavia terricola*) exhibited the highest IAA production at 6.40 mg/L and 6.61 mg/L, respectively. The strain LF2 (*Penicillium oxalicum*) demonstrated the highest siderophore producing and phosphate-solubilizing abilities, with SU activity at 61.74% and phosphorolytic activity at 9.97 mmol/L. The fungus LF4 (*Aspergillus* sp.) displayed catalase (antioxidative) activity at 64.31 μmol/mL (Table 4). Based on these results, the latter four strains (FL1, RT1, LF2, and LF4) were selected for co-cultivation with seedlings of *A. orientale* to observe their effects on plant growth and triterpenoid accumulation.

**Table 4:** Quantitative detection of growth promoting abilities of endophytic fungi.

Strains	IAA (mg/L)	SU (%)	CAT (μmol/mL)	phosphorus (mmol/L)
FL1	6.40 ± 0.48	11.47 ± 0.31	—	—
FL3	1.46 ± 0.02	—	21.95 ± 0.31	—
LF1	2.44 ± 0.25	—	26.81 ± 0.39	—
LF2	—	61.74 ± 0.49	—	9.97 ± 0.51
LF3	0.36 ± 0.02	—	—	0.38 ± 0.02
LF4	—	22.68 ± 0.16	64.31 ± 0.14	9.18 ± 0.49
LF5	1.98 ± 0.02	—	—	—
RH1	—	—	0.72 ± 0.03	0.58 ± 0.11
RH6	4.64 ± 0.39	7.36 ± 0.10	22.81 ± 0.94	6.24 ± 0.87
RT1	6.61 ± 0.23	8.62 ± 0.14	3.71 ± 0.51	—

Values were presented as the means ± SD (*n* = 3).

### 3.4 Co-cultivation of select endophytic fungal isolates with *A. orientale*

**Figure 5:**
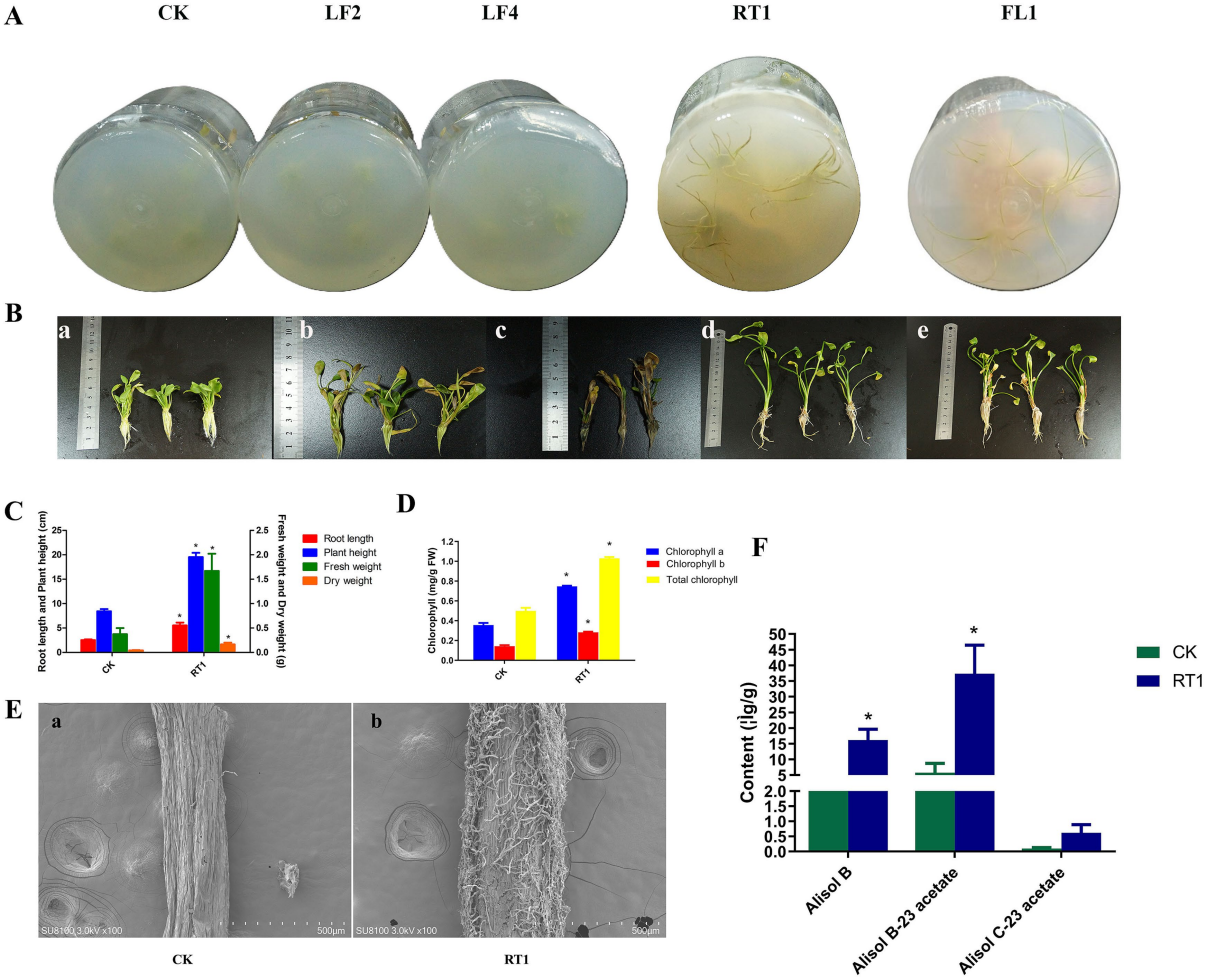
**(A-B)** Growth status of *A. orientale* seedlings after co-cultivated with select endophytic fungal isolates, a CK group; b LF2 group; c LF4 group; d RT1 group; e FL1 group. **(C)** Effects of RT1 on root length, plant height, fresh weight and dry weight of *A. orientale*. **(D)** Chlorophyll content of *A. orientale* in different treatment groups. **(E)** Colonization of RT1 in the roots of *A. orientale* with SEM analysis, a control (CK) treatment; b *P. terricola* (RT1) treatment. **(F)** The content of triterpenoids in *A. orientale* after treatment with RT1. * *p* < 0.05 vs. the CK group, *n* = 3.

The effects of isolates *Nigrospora sphaerica* (FL1), *Psedothielavia terricola* (RT1), *Penicillium oxalicum* (LF2), and *Aspergillus* sp. (LF4) on growth and/or triterpenoid accumulation in *A. orientale* were examined in co-cultivation experiments as detailed in the Methods section. After 3 weeks of co-cultivation, plants inoculated with RT1 demonstrated increased height and longer roots compared to control (CK) treatments; similar findings were observed in plants that were inoculated with FL1. However, inoculation treatments with LF2 and LF4 inhibited the growth of *A. orientale* as compared to the control (Figures 5A,B). Based on these results, isolate RT1 was used for further investigation.

### 3.5 Effect of *Psedothielavia terricola* (RT1) on *A. orientale* growth

Further in-depth assessment of the effects of *P. terricola* on *A. orientale* revealed enhanced growth and increased root architecture effects. Compared to the control plants, those inoculated with RT1 showed 103 and 121% increases in root length and plant height, respectively. Moreover, a 3–3.5 fold increase in fresh weight and dry weight was observed, coupled to a significant (*p* < 0.01) increase in chlorophyll content (Figures 5C,D). However, no significant difference was found in the number of leaves and the number of lateral roots between RT1-treated and control plants. Mycelial colonization of plant roots was monitored by scanning electron microscopy (SEM). The results confirmed proliferation of RT1 hyphae within *A. orientale* roots (Figure 5E).

### 3.6 Effects of *P. terricola* (RT1) on the accumulation of triterpenoids in *A. orientale*

Alisol B-23 acetate, alisol C-23 acetate and alisol B are three main triterpenoid compounds found in *A. orientale* and are essential for its pharmacological efficacy. In plants co-cultured with RT1, the contents of alisol B-23 acetate, alisol C-23 acetate and alisol B were 5.50, 5.20, and 4.42 times higher than those of control treatments, respectively (Figure 5F).

### 3.7 Transcriptome analysis of the *P. terricola* (RT1)-*A. orientale* interaction

To elucidate the effects of RT1 on *A. orientale* global gene expression, transcriptome sequencing was performed to examine changes in gene expression. A total of ten samples were prepared, consisting of five samples (plants) inoculated with RT1 and five control (CK) samples. A total of 514,552,178 clean reads were obtained, with a total nucleotide count of 7.74 Gb, and a GC content of 53.42%. Following *de novo* assembly, 79,874 unigenes were generated, with a total length of 98.18 Mb, an average length of 1,288 bp, and an N50 length of 2,171 bp. Among the total set of unigenes, 35,325 unigenes (44.23%) ranged from 200 to 500 bp, 16,885 (21.14%) ranged from 500 to 1,000 bp, and 27,664 (34.64%) exceeded 1,000 bp in length (Supplementary Figure S2).

**Figure 6:**
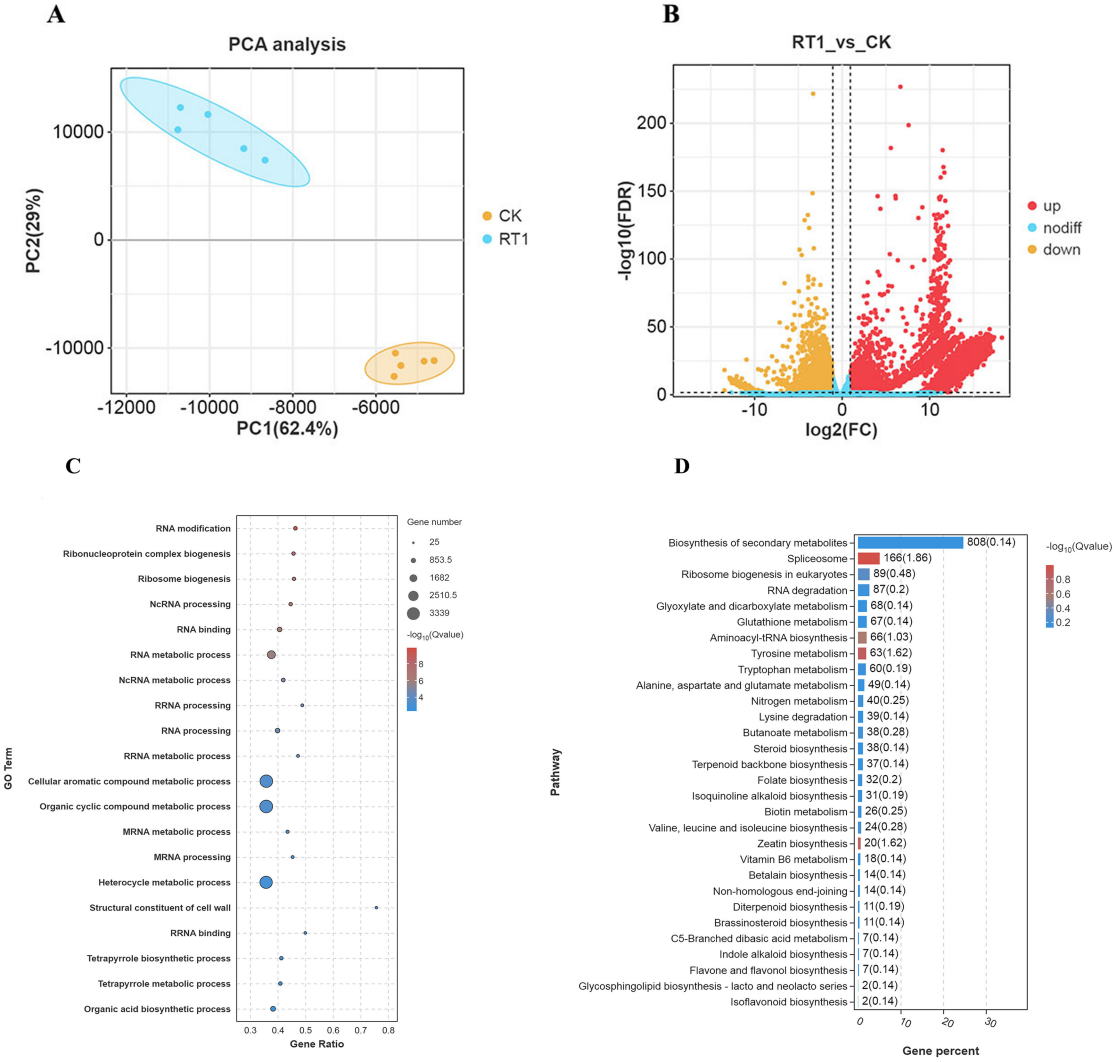
Transcriptome analysis of the *P. terricola* (RT1)-*A. orientale* interaction. **(A)** PCA analysis of DEGs for RT1 and CK groups. **(B)** Volcano plot of DEGs for RT1 and CK groups. **(C)** GO terms of RT1_vs_CK. **(D)** KEGG pathway enrichment analyses of RT1_vs_CK.

Principal component analyses showed a clear distinction in global gene expression between RT1- treated and control plants. Replicates, especially those from RT1-treated plants, demonstrated tight clustering (Figure 6A). The analysis identified 10,809 up-regulated differentially expressed genes (DEGs) and 2,521 down-regulated DEGs, as shown in the volcano plots in Figure 6B.

GO and KEGG enrichment analyses were performed to analyze the DEGs, as detailed in the Methods section. The top 20 enriched GO terms following RT1 treatment including “organic cyclic compound metabolic process,” “cellular aromatic compound metabolic process,” and “heterocycle metabolic process,” were all involved in metabolite programming (Figure 6C). Similarly, KEGG enrichment analysis revealed pathways associated with growth promotion during fungal co-culture, including “biosynthesis of secondary metabolites,” “indole alkaloid biosynthesis,” and “terpenoid backbone biosynthesis” (Figure 6D).

KEGG pathway annotation revealed that 3,268 DEGs were enriched in 140 relevant pathways in the RT1 group compared to the control group (Table 5). These included “terpenoid backbone biosynthesis,” “indole alkaloid biosynthesis,” “sesquiterpenoid and triterpenoid biosynthesis,” “phenylalanine, tyrosine and tryptophan biosynthesis,” “photosynthesis,” and “oxidative phosphorylation.” Within the “terpenoid backbone biosynthesis” and “sesquiterpenoid and triterpenoid biosynthesis” pathways, several key genes were significantly upregulated in the RT1 group, including 3-hydroxy-3-methylglutaryl-CoA reductase (HMGCR), mevalonate diphosphate decarboxylase (MVD), farnesyl diphosphate synthase (FPPS), 1-deoxy-D-xylulose-5-phosphate reductoisomerase (DXR), 1-deoxy-D-xylulose-5-phosphate synthase (DXS), farnesyl-diphosphate farnesyltransferase (FDFT1), and squalene monooxygenase (SQLE). With respect to the “indole alkaloid biosynthesis” and “phenylalanine, tyrosine and tryptophan biosynthesis” pathways, the RT1-treated group showed significant upregulation of the gene expression levels of aromatic-L-amino-acid/L-tryptophan decarboxylase (TDC2), indole-3-glycerol phosphate synthase (TRPC), phosphoribosylanthranilate isomerase (TRP1), and tryptophan synthase (TRP). In the “oxidative phosphorylation” pathway, the expression levels of cytochrome c oxidase subunit 1 (COX1), cytochrome c oxidase subunit 10 (COX10) cytochrome c oxidase assembly protein subunit 11 (COX11), cytochrome c oxidase subunit 5b (COX5B), NADH dehydrogenase (ubiquinone) Fe-S protein 1 (NDUFS1), and cytochrome c (CYC) genes were significantly upregulated in RT1-treated group. Furthermore, in the “photosynthesis” pathway, genes related to photosynthesis (e.g., photosystem I subunit IV (PSAE), light-harvesting complex II chlorophyll a/b binding protein 1 (LHCB1), and light-harvesting complex II chlorophyll a/b binding protein 6 (LHCB6) were upregulated in the RT1 group).

**Table 5:** Changes of DEGs related to growth promotion after RT1 intervention.

Pathway (ID)	Unigene ID	Symbol	Description	Log2(fc)
Terpenoid backbone biosynthesis (ko00900)	Unigene0072115	HMGR	hydroxymethylglutaryl-CoA reductase (NADPH)	15.42
	Unigene0025467	MVD	diphosphomevalonate decarboxylase	14.64
	Unigene0075775	FPPS	farnesyl diphosphate synthase	9.25
	Unigene0037438	DXR	1-deoxy-D-xylulose-5-phosphate reductoisomerase	1.15
	Unigene0055691	DXS	1-deoxy-D-xylulose-5-phosphate synthase	2.54
Sesquiterpenoid and triterpenoid biosynthesis (ko00909)	Unigene0074979	FDFT1	farnesyl-diphosphate farnesyltransferase	14.81
	Unigene0080344	SQLE	squalene monooxygenase	14.40
Indole alkaloid biosynthesis (ko00901)	Unigene0003272	TDC2	aromatic-L-amino-acid/L-tryptophan decarboxylase	4.32
Phenylalanine, tyrosine and tryptophan biosynthesis (ko00400)	Unigene0085841	TRPC	indole-3-glycerol phosphate synthase	1.07
	Unigene0031683	TRP1	anthranilate synthase / indole-3-glycerol phosphate synthase / phosphoribosylanthranilate isomerase	12.51
	Unigene0047272	TRP	tryptophan synthase	15.17
Phenylalanine metabolism (ko00360)	Unigene0016509	PAL	phenylalanine ammonia-lyase	1.41
	Unigene0058165	ECHA	enoyl-CoA hydratase	12.24
Phenylpropanoid biosynthesis (ko00940)	Unigene0060391	CCR	cinnamoyl-CoA reductase	1.61
	Unigene0012542	CYP73A	trans-cinnamate 4-monooxygenase	2.45
Photosynthesis (ko00195)	Unigene0009630	PSAE	photosystem I subunit IV	1.13
Photosynthesis - antenna proteins (ko00196)	Unigene0064578	LHCB1	light-harvesting complex II chlorophyll a/b binding protein 1	1.80
	Unigene0049297	LHCB6	light-harvesting complex II chlorophyll a/b binding protein 6	1.40
Oxidative phosphorylation (ko00190)	Unigene0016666	COX1	cytochrome c oxidase subunit 1	10.28
	Unigene0008330	COX10	heme o synthase	13.50
	Unigene0083961	COX11	cytochrome c oxidase assembly protein subunit 11	9.08
	Unigene0038385	COX5B	cytochrome c oxidase subunit 5b	15.82
	Unigene0056976	NDUFS1	NADH dehydrogenase (ubiquinone) Fe-S protein 1	15.00
	Unigene0063923	CYC	cytochrome c	15.58

**Figure 7:**
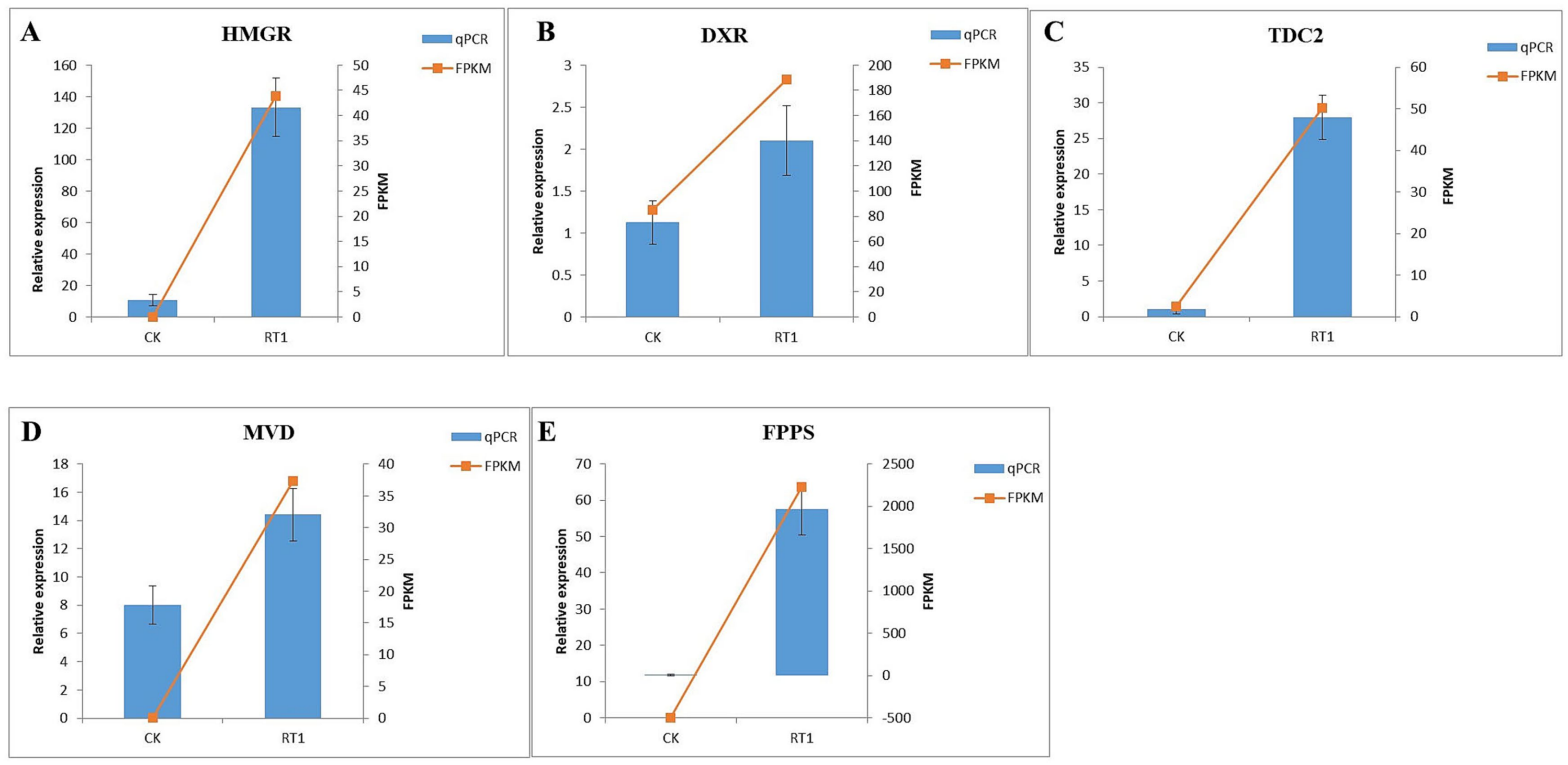
Verification of transcriptome sequencing data by RT-qPCR (x̄ ± SD, n = 3). **(A)** HMGR; **(B)** DXR; **(C)** TDC2; **(D)** MVD; **(E)** FPPS.

To validate the transcriptomics data, RT-*q*PCR was conducted on select genes. Five DEGs (HMGR, DXR, TDC2, MVD, and FPPS) associated with growth promotion and triterpenoid accumulation were selected for expression verification, showing high correlation with the RNA-seq data (Figure 7).

### 3.8 Whole-genome sequencing of *P. terricola* (RT1)

**Figure 8:**
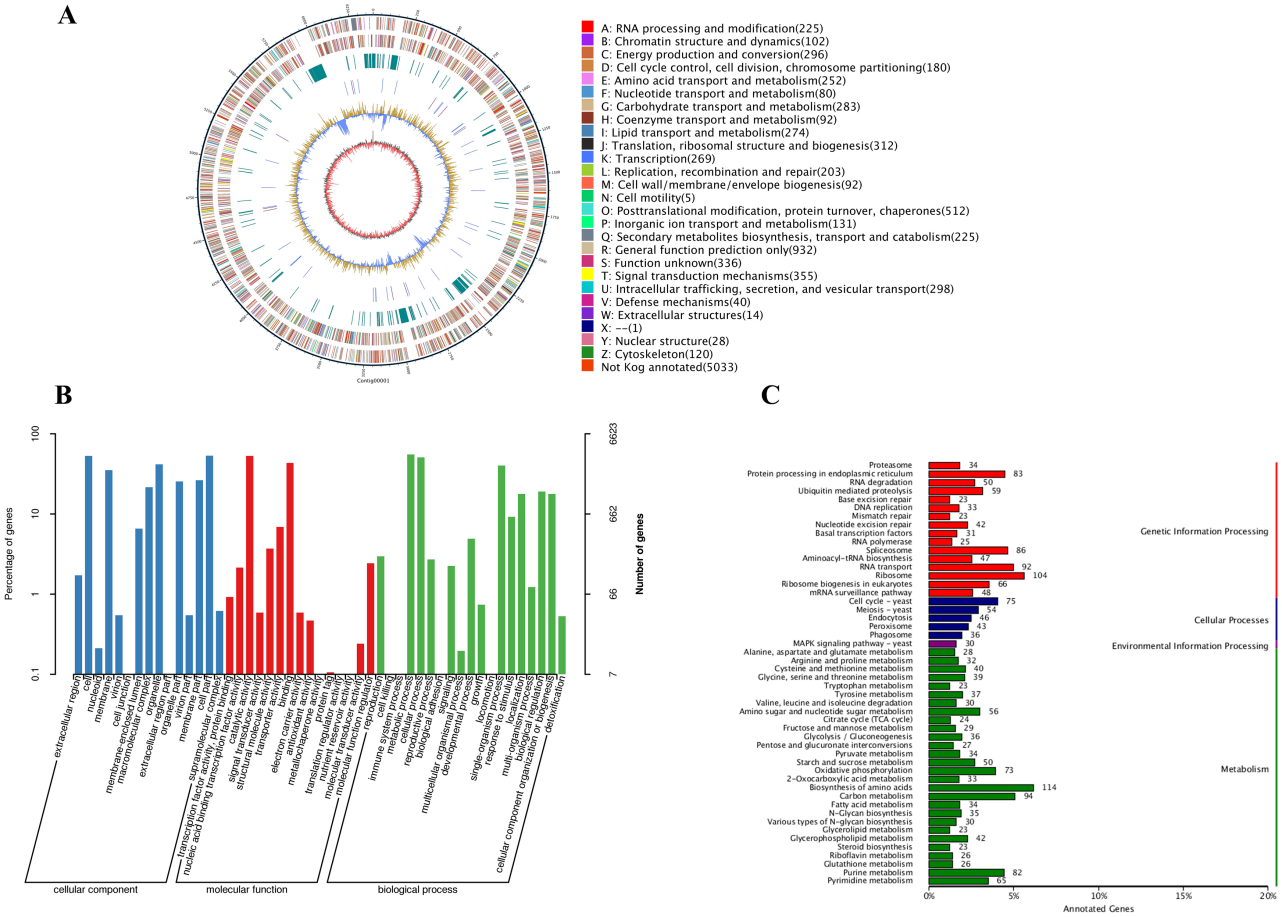
Whole-genome sequencing of *P. terricola* (RT1). **(A)** RT1 genome circle map. The outermost circle illustrates the genome size. The second and third circles represent the coding sequences (CDS) on the positive and negative strands, respectively. The fourth circle is designated for repetitive sequences, the fifth circle indicates rRNA and tRNA, the sixth circle displays the GC content, and the innermost circle demonstrates GC skew. **(B)** GO classifications of predicted genes in RT1. **(C)** KEGG pathway enrichment analyses of RT1.

To investigate the molecular mechanisms underlying the endophytic interaction between *P. terricola* (RT1) and *A. orientale*, the genome of the fungus was analyzed. The general genome features of *P. terricola* (RT1) are shown in Table 6 and Supplementary Figure S3. The *P. terricola* (RT1) genome was found to comprise 34.5 Mb with a GC content of 57.19%, and could be assembled into 25 contigs (N50 size: 4.98 Mb), with contig 1 being the longest (Figure 8A). Genome assembly quality was supported by the BUSCO analysis, showing 95.86% completeness. A summary of the key characteristics is provided in Table 6, including 10,054 protein-coding genes with an average length of 1,736 bp, 403 tRNAs, 85 rRNAs (5S, 18S, 5S, and 28S), and 38 other non-coding RNAs.

**Table 6:** Whole-genome assembly results of RT1.

Assembly parameters	Value
Genome size (bp)	34,546,314
GC content (%)	57.19%
BUSCO (%)	95.86%
Number of contigs	25
Contig N50 (bp)	4,981,977
Number of Genes	10,580
Number of protein-coding genes	10,054
Total gene length (bp)	17,460,547
Average gene length (bp)	1,736.68
tRNAs	403
5.8S rRNA	11
18S rRNA	21
28S rRNA	11
5S rRNA	42
Other ncRNA	38

The 10,054 predicted genes were blasted against GO, Nr, Swissprot, TrEMBL, KEGG, KOG, and Pfam databases, with 6,623 predicted genes receiving a GO assignment (Figure 8B). The three categories of annotations (cellular component, molecular function, and biological process) contained 17,667, 7,590, and 14,920 GO terms. A total of 3,656 genes were classified within the metabolic process category. The KEGG pathway analysis revealed a total of 3,163 genes that were annotated within four metabolic pathways, specifically genetic information processing (846), cellular processes (254), environmental information processing (30), and metabolism (1185) (Figure 8C). In the process of metabolism, a total of 114 genes were primarily involved in amino acid biosynthesis.

**Figure 9:**
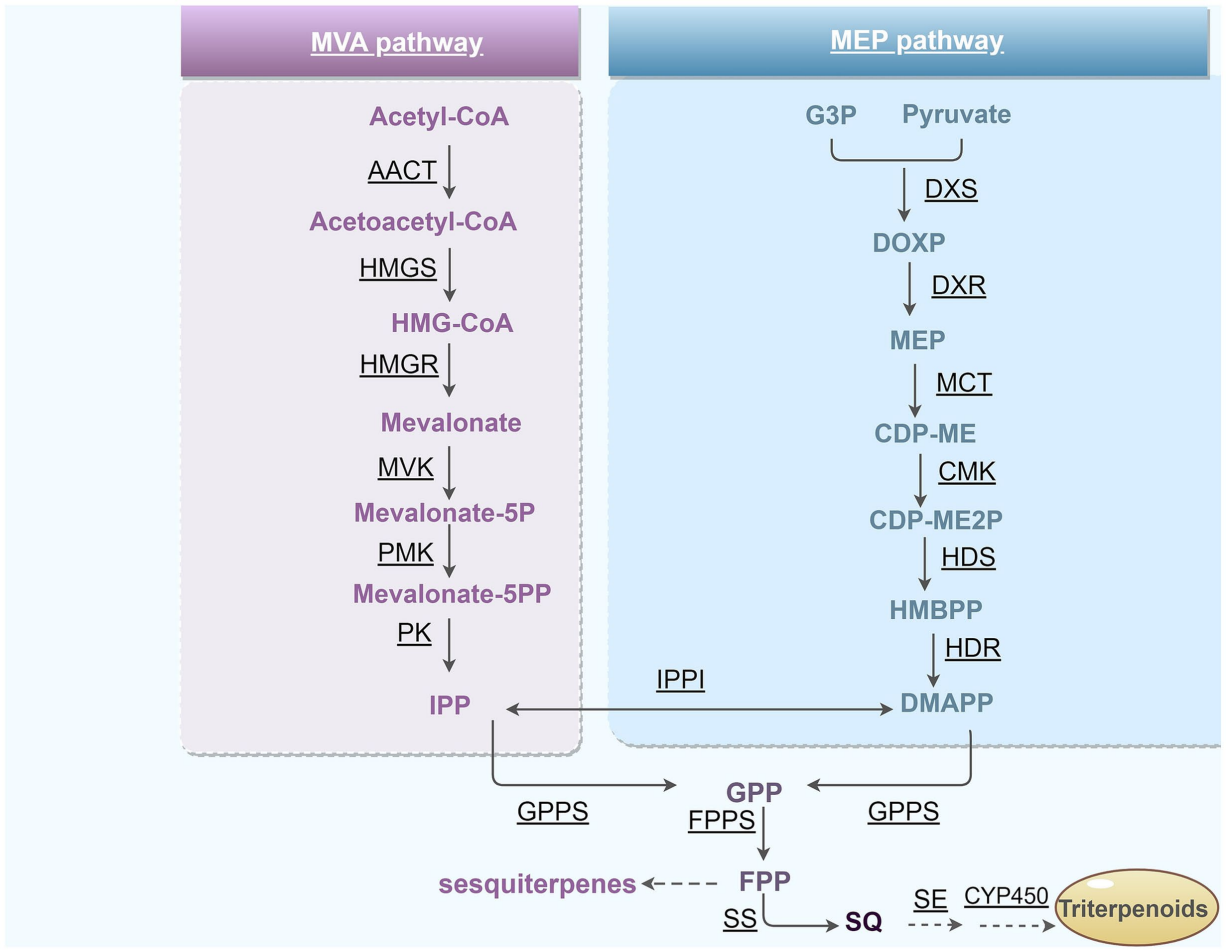
MVA and MEP/DOXP pathways in triterpenoid skeleton biosynthesis.

Additionally, a set of genes encoding for enzymes involved in terpenoid backbone biosynthesis was identified. The KEGG analysis indicated that genes involved in triterpenoids biosynthesis were annotated within the “terpenoid backbone biosynthesis” (ko00900) and “sesquiterpenoid and triterpenoid biosynthesis” (ko00909) pathways. These pathways included the enzymes acetyl-CoA C-acetyltransferase (ACAT), hydroxymethylglutaryl-CoA synthase (HMGCS), hydroxymethylglutaryl-CoA reductase (NADPH) (HMGCR), mevalonate kinase (MVK), diphosphomevalonate decarboxylase (MVD), squalene epoxidase (SQLE), and farnesyl-diphosphate farnesyltransferase (FDFT1) (Supplementary Figures S4, S5). A summary of potential key genes involved in triterpenoid synthesis was displayed (Figure 9). Ko00400 is the major biosynthesis pathway for tryptophan, which is precursor of indole-3-acetic acid (IAA). Moreover, genes involved in tryptophan biosynthesis, including *trpA*, *trpD* and *trp1*, were detected in the *P. terricola* genome (Table 7).

**Table 7:** Genes potentially associated with triterpenoid accumulation and growth promotion in the genome of RT1.

Function/Pathway	Gene ID	Symbol	Description	Location/Locus tag
Terpenoid backbone biosynthesis (ko00900)	EVM0G036120	HMGCR	hydroxymethylglutaryl-CoA reductase (NADPH)	Contig3:4061530: 4065120
	EVM0G024380	MVD	diphosphomevalonate decarboxylase	Contig3: 102649: 103964
	EVM0G077730	ACAT	acetyl-CoA C-acetyltransferase	Contig6: 2439617: 2441028
	EVM0G029250	HMGCS	hydroxymethylglutaryl-CoA synthase	Contig3: 1860896: 1862737
	EVM0G004220	MVK	mevalonate kinase	Contig1: 1390729: 1392348
Sesquiterpenoid and triterpenoid biosynthesis (ko00909)	EVM0G098900	FDFT1	farnesyl-diphosphate farnesyltransferase	Contig8: 1273395: 1275022
	EVM0G079850	SQLE	squalene epoxidase	Contig6: 3143408: 3144934
Tryptophan biosynthesis (ko00400)	EVM0G094880	TRPA	tryptophan synthase	Contig7: 3359254: 3361593
	EVM0G077180	TRPD	anthranilate phosphoribosyltransferase	Contig6: 2273053: 2274365
	EVM0G066780	TRP1	prephenate dehydrogenase (NADP+)	Contig5: 3862562: 3864933

## 4 Discussion

Endophytic fungi engage in mutualistic relationships with host plants, significantly influencing plant productivity and health (Yan et al., 2019; Shen et al., 2023). The composition and structure of endophytic fungi vary across plant tissues, with some species being tissue-specific. This tissue specificity plays a crucial role in the accumulation of bioactive compounds in different plant tissues (Fan M. et al., 2020; Fan S. et al., 2020). Considering its medicinal importance, elucidating the roles of endophytic fungi and their effects on growth and bioactive compound accumulation may enhance the value of the crop. This study characterized endophytic fungi from different parts of the *A. orientale* plant, including its rhizome, root, leaves, and flower, combining ITS amplicon sequencing with culturing approaches. The endophytic fungal community in *A. orientale* was strongly influenced by tissue type. Fungal abundance was highest in the rhizome, followed by flowers, roots, and leaves; in contrast, the overall diversity was highest in the rhizome, followed by roots, flowers, and leaves. Members of the genera *Penicillium, Cladosporium*, and *Plectosphaerella* predominated in the rhizome, *Cladosporium*, *Aspergillus*, *Alternaria*, and *Moesziomyces* in the roots, *Cladosporium* and *Malassezia* in the flowers, and *Plectosphaerella*, *Rhizopus*, and *Chaetomium* in the leaves, indicating a wide variation in different parts of the *A. orientale* at the genus level.

Fungal endophytes have been shown to play an essential role in growth and/or secondary metabolite stimulation in various medicinal plants (Tsipinana et al., 2023). For example, the endophytic fungus *Mucor circinelloides* DF20 can stimulate the growth and induce tanshinone biosynthesis and accumulation in the Red sage (*Salvia miltiorrhiza*) root (Chen et al., 2021). Endophytic fungi can regulate a range of active compounds in medicinal plants, including those involved in resistance to environmental stresses and promoting the accumulation of valuable metabolites (Zhang et al., 2024). Endophytes promote plant growth and facilitate nitrogen fixation, phosphorus solubilization, siderophore production, and improve oxidation resistance, as well as the synthesis of plant hormones such as ethylene (Bajpai and Johri, 2019; Khan et al., 2020; Ibrahim et al., 2021; Ding et al., 2022). A total of 19 endophytic fungal isolates from the various *A. orientale* plant parts were cultured and identified by their morphological characterizations (Figure 3), combined with molecular sequence analyses of ITS/LSU genes. Moreover, in conjunction with the relevant fungal genera, further investigation of genes, including ITS, LSU, IGS, TEF, CAM, RPB1, RPB2, TUB2, ACT, CMD, GAPDH, HIS, TEF1-*α*, and BenA, was conducted to confirm the species classification by detecting multiple genes (Supplementary Table S3). Two promising candidates, *P. terricola* (RT1) and *Nigrospora sphaerica* (FL1), were identified. Based on further testing, isolate *P. terricola* (RT1), from roots of *A. orientale*, was shown to possess the potential to produce IAA at 6.61 mg/L (Table 4). The production of IAA in *P. terricola* (RT1) could be an important factor contributing to plant growth promotion. In addition, the annotated *P. terricola* (RT1) genome also revealed the presence of several gene clusters involved in tryptophan synthesis, such as *trpA*, *trpD* and *trp*1. In plant growth-promoting microbes, gene clusters involved in phytohormone pathways are commonly present, particularly those related to IAA production (Abdullahi et al., 2021; Yang et al., 2023). To better understand the growth promoting effects of *P. terricola* (RT1), further studies are warranted.

Triterpenoids are natural compounds widely distributed in nature, known for their remarkable biological activities. Triterpenoids in *A. orientale* were reported to be responsible for its versatile pharmacological activities (Gao et al., 2018). The majority of triterpenoids are the result of specialized metabolism in plants. Microbial biosynthesis of triterpenoids can minimize the need for cultivating, harvesting, and extracting plant material. Simultaneously, it offers an eco-friendly synthesis platform for specialized terpenoids, enabling their high-yield and high-purity production (Belcher et al., 2020). Some endophytic fungi can produce metabolites similar to, or even identical to, those produced by their host plants (Gao et al., 2025). In this study, only two strains, RT1 and FL1, could be long-term co-cultured with *A. orientale* seedlings without causing obvious disease symptoms and significantly increased the biomass of *A. orientale*. Seedlings of *A. orientale* co-cultivated with FL1 led to a minor increase in the accumulation of triterpenoids compared to the control group, although this increase was not statistically significant (data not shown). The contents of three triterpenoids were enhanced by *P. terricola* (RT1) inoculation compared with those of the control plants, which indicated that RT1 may activate the biosynthetic pathway of triterpenoids in the whole *A. orientale* plant. These findings were supported by transcriptomic analyses, which indicated fungal-dependent activation of plant terpenoid and tryptophan biosynthesis, as well as enhanced expression of genes involved in photosynthesis and oxidative phosphorylation, correlating with the plant growth promotion and triterpenoid accumulation. These changes were aligned with the findings of previous studies (Silva et al., 2020; Yang et al., 2023).

In conclusion, the diversity of fungal endophytes was examined across different tissues of the medicinal plant *A. orientale*. Two promising fungal strains were isolated, capable of promoting plant growth. One such fungal strain, characterized as *P. terricola*, was found to display high growth promotion and stimulation of triterpenoids during co-cultivation with *A. orientale*. The genome sequence of the fungus was determined, and compatible/parallel (with the plant) pathways for triterpenoid synthesis were annotated. Transcriptomics analyses showed induction of plant growth-promoting and triterpenoid biosynthetic pathways/factors during co-cultivation, i.e., presumably during endophytic colonization of *A. orientale* by *P. terricola*. Collectively, these findings support the use of combined screening and validation approaches to identify fungal (endophyte) partners of medicinal plants that enhance plant growth and bioactive compound accumulation, thereby increasing crop value and utility.

## Data Availability

The datasets generated during the current study were deposited and are available at the National Center for Biotechnology Information (NCBI) public database. All ITS rRNA gene, transcriptome analysis and whole-genome sequence analysis raw sequences can be found in Sequence Read Archive (SRA) under BioProject no. PRJNA1299686, PRJNA1348109 and PRJNA1347775. Other data generated or analyzed during this study were included in this published article and its additional files.
