# Supplementary material for: A fungal endophyte of the medicinal plant, Alisma orientale, promotes plant growth and bioactive compound accumulation
Source: Front Microbiol. 2026 Mar 3;17:1773907. doi: 10.3389/fmicb.2026.1773907 (PMC12992292; doi:10.3389/fmicb.2026.1773907)
Supplement: Supplementary file 1 [file Table_1.docx]

Supplementary Material

# Supplementary Figures and Tables

## Supplementary Tables

Supplementary Table S1. Primers pair used for amplification of target genes

| Genes | Primers pair | Primers sequence (5'-3) | Reference |
| --- | --- | --- | --- |
| ITS | ITS1 | TCCGTAGGTGAACCTGCGG | White et al. (1990) |
|  | ITS4 | TCCTCCGCTTATTGATATGC |  |
| LSU | LROR | GTACCCGCTGAACTTAAGC | Vilgalys et al. (1990) |
|  | LR5 | ATCCTGAGGGAAACTTC |  |
| IGS | iNL11 | AGGCTTCGGCTTAGCGTCTTAG | Epstein et al. (2017) |
|  | FOIGS-R | GCCGACACCGCGCCTCTTAA |  |
| TEF1 | EF1 | ATGGGTAAGGARGACAAGAC | O'Donnell et al. (1998) |
|  | EF2 | GGARGTACCAGTSATCATG |  |
| CAM | CL1 | GARTWCAAGGAGGCCTTCTC | O'Donnell et al. (2000) |
|  | CL2A | TTTTTGCATCATGAGTTGGAC |  |
| RPB1 | RPB1-F | CTGTTCGCAACTCTCTGGGT | This study |
|  | RPB1-R | TCCGGATGCGAGCATTTACC |  |
| RPB2 | fRPB2-5F-nd | GACGACCGTGATCATTTCGG | Vaghefi et al. (2021) |
|  | fRPB2-414R-nd | ACAAGTCCCCAATGCGTATTGTG |  |
| TUB2 | T1 | AACATGCGTGAGATTGTAAGT | O'Donnell et al. (1997) |
|  | T2 | TAGTGACCCTTGGCCCAGTTG |  |
| ACT | ACT-512F | TGT GCA AGG CCG GTT TCG C | Carbone et al. (1999) |
|  | ACT-783R | TAC GAG TCC TTC TGG CCC AT |  |
| CMD | CAL228F | GAG TTC AAG GAG GCC TTC TCC C | Groenewald et al. (2013) |
|  | CAL2Rd | TGR TCN GCC TCD CGG ATC ATC TC |  |
| GAPDH | Gpd1-LM | ATT GGC CGC ATC GTC TTC CGC AA | Myllys et al. (2002) |
|  | Gpd2-LM | CCC ACT CGT TGT CGT ACC A |  |
| HIS | CYLH3F | AGG TCC ACT GGT GGC AAG | Crous et al. (2002) |
|  | CYLH3R | AGC TGG ATG TCC TTG GAC TG |  |
| TEF1-α | EF1-728F | CAT CGA GAA GTT CGA GAA GG | Carbone et al. (1999) |
|  | EF1-986R | TAC TTG AAG GAA CCC TTA CC |  |
| BenA(β-Tubulin) | Bt2a | GGTAACCAAATCGGTGCTGCTTTC | Lousie et al. (1995) |
|  | Bt2b | ACCCTCAGTGTAGTGACCCTTGGC |  |

Supplementary Table S2. The primer information for qPCR

| Genes | Primers pair | Primers sequence (5'-3) | Product size (bp) |
| --- | --- | --- | --- |
| HMGR | HMGR-F | AACTCGTTCACCACCTACTCG | 137 |
|  | HMGR-R | CCATGTTCGGTCTCCCTC | 137 |
| DXR | DXR-F | TCTGCTATCTTCCAGTGTATCC | 163 |
|  | DXR-R | TCTTCTTGCCCATGTTCC | 163 |
| TDC2 | TDC2-F | TGGGGTCAGTTGTTTTGC | 184 |
|  | TDC2-R | CAGTCCATCACGAGGTTTTC | 184 |
| FPPS | FPPS-F | GCTCGATTGGTTCAAGAATG | 224 |
|  | FPPS-R | GCTGGTCACGACGGGTAA | 224 |
| UBC9 | UBC9-F | GTCTTCCTTGTCACCATTCAT | 140 |
|  | UBC9-R | GGGCTCCATTGTTCCTTT | 140 |
| MVD | MVD -F | CGCCCTTATCCTCGTCGTC | 102 |
|  | MVD -R | GCGATGCGTTGCTGGAAG | 102 |

Supplementary Table S3. Identification of endophytic fungi species by combination of multiple genes

| LOCUS | ITS | LSU | IGS | TEF1 | CAM | RPB1 | RPB2 | TUB2 | ACT | CMD | GAPDH | HIS | TEF-α | BenA |
| --- | --- | --- | --- | --- | --- | --- | --- | --- | --- | --- | --- | --- | --- | --- |
| *Penicillium* | √ | √ |  |  | √ |  | √ |  |  |  |  |  |  | √ |
| *Nigrospora* | √ | √ |  |  |  |  | √ |  |  |  |  |  | √ |  |
| *Cercospora* | √ | √ |  |  |  |  | √ | √ | √ | √ | √ | √ | √ |  |
| *Cladosporium* | √ | √ |  | √ |  |  |  |  | √ |  |  |  |  |  |
| *Aspergillus* | √ | √ |  |  | √ |  | √ |  |  |  |  |  |  | √ |
| *Talaromyces* | √ | √ |  |  |  |  |  |  |  |  |  |  |  |  |
| *Fusarium* | √ | √ | √ | √ | √ | √ | √ | √ |  |  |  |  |  |  |
| *Pestalotiopsis* | √ | √ |  | √ |  |  |  | √ |  |  |  |  |  |  |
| *Pseudothielavia* | √ | √ |  |  |  |  | √ | √ |  |  |  |  |  |  |

## Supplementary Figures


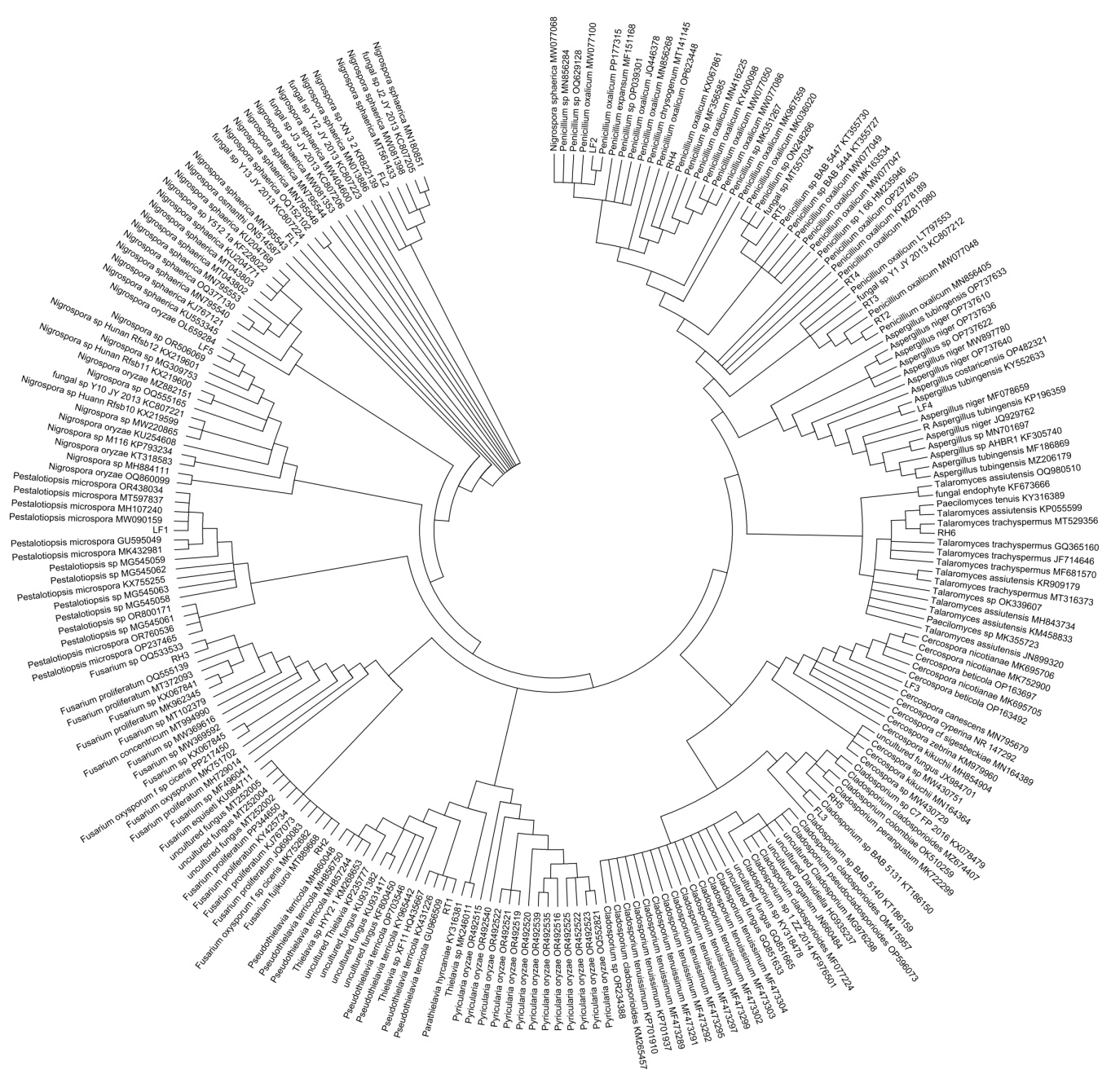


Supplementary Figure S1. The phylogenetic tree of 19 endophytic fungi.

**
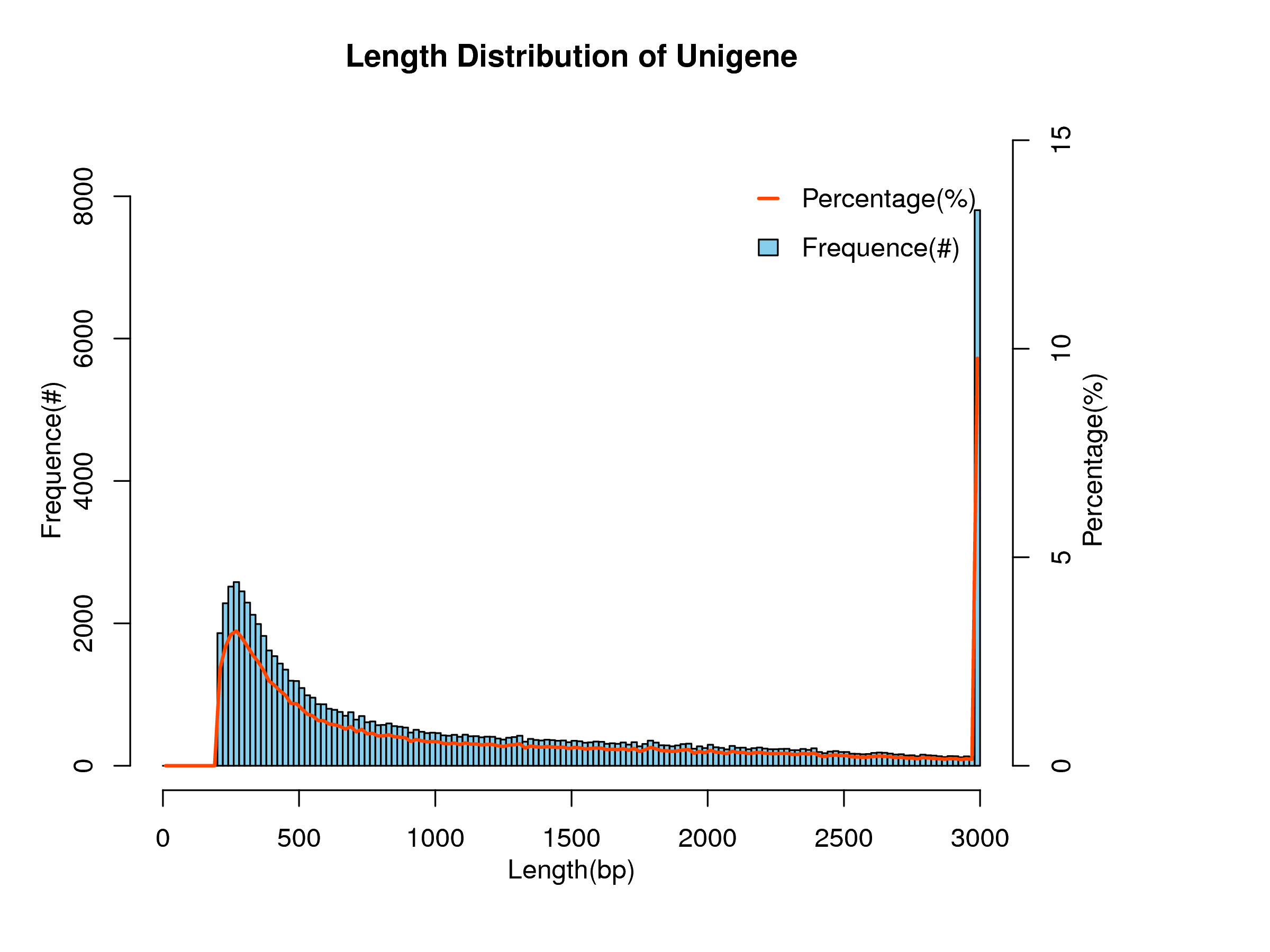
**

Supplementary Figure S2. Length distribution of unigenes for *A. orientale* after inoculated with *P. terricola* (RT1).

**
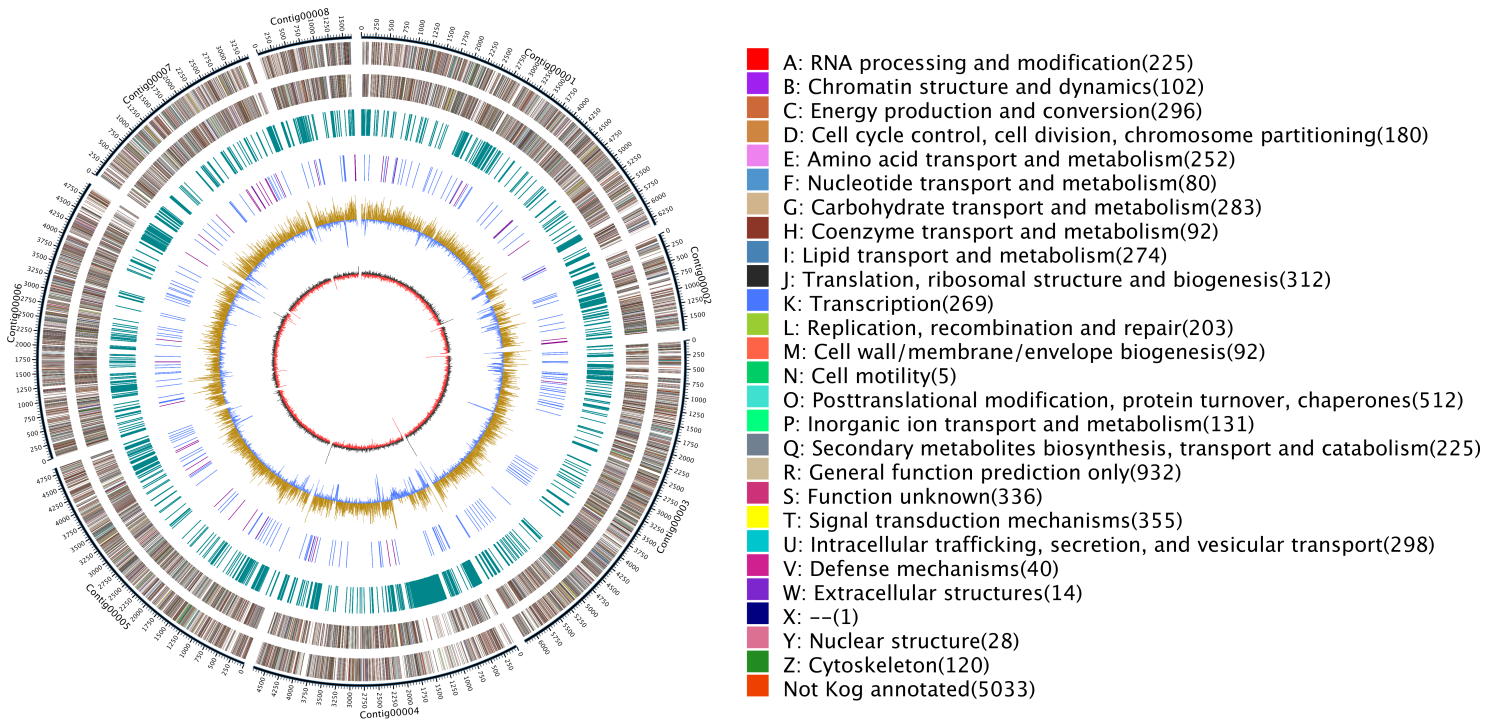
**

Supplementary Figure S3. Circular representation of *P. terricola* (RT1) chromosome. The outermost circle illustrates the genome size. The second and third circles represent the coding sequences (CDS) on the positive and negative strands, respectively. The fourth circle is designated for repetitive sequences, the fifth circle indicates rRNA and tRNA, the sixth circle displays the GC content, and the innermost circle demonstrates GC skew.


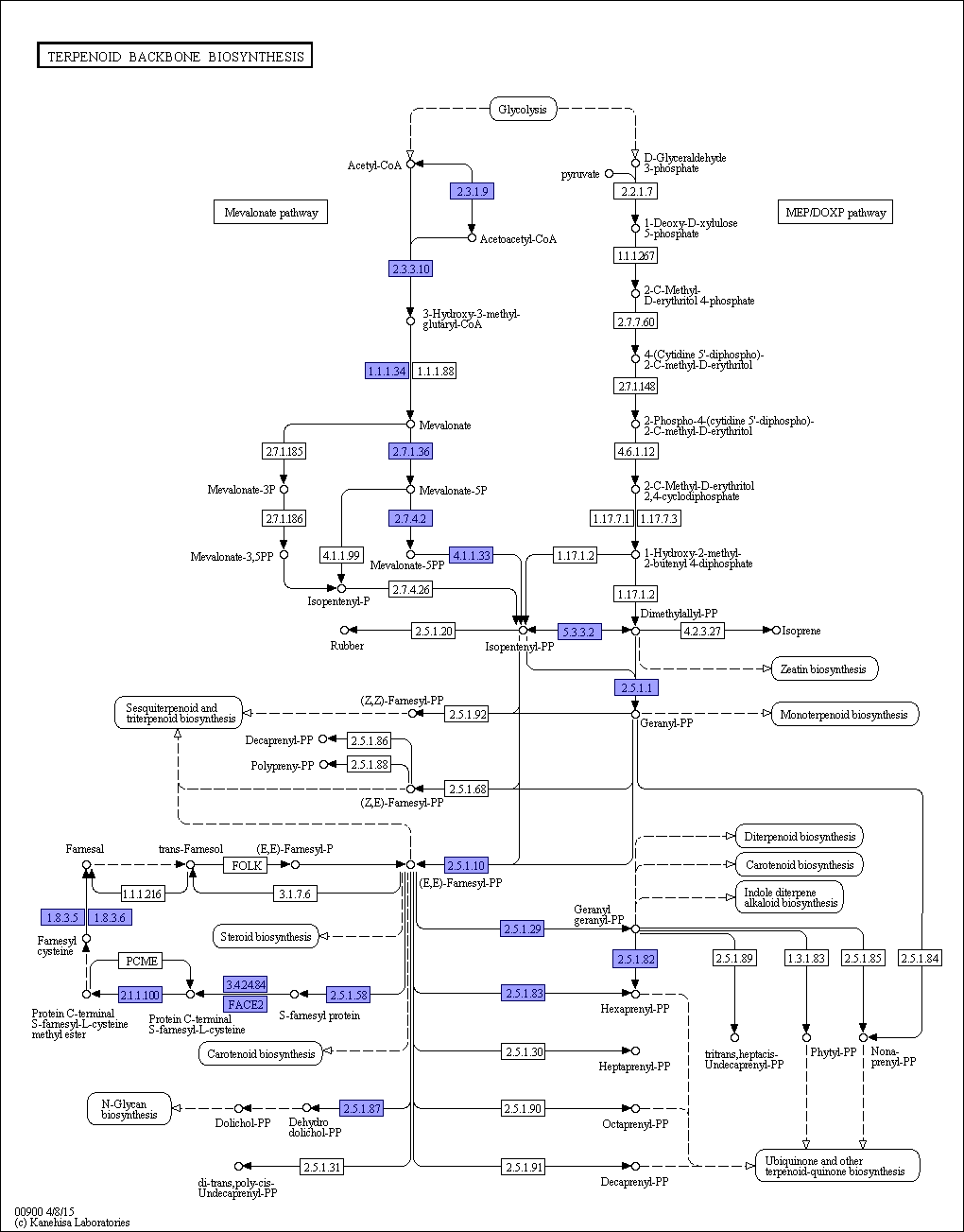


Supplementary Figure S4. Biosynthetic pathway of terpenoid backbone.


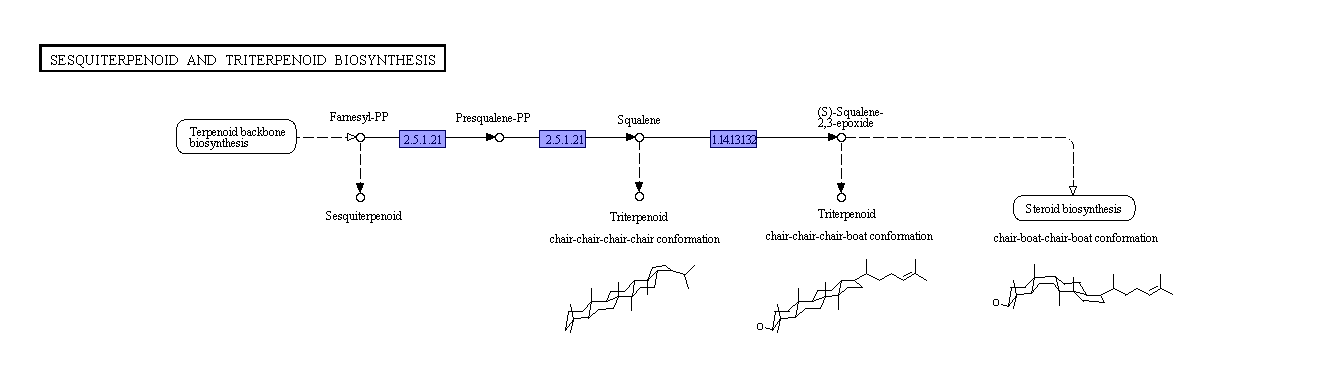


Supplementary Figure S5. Biosynthetic pathway of sesquiterpenoid and triterpenoid.
